# Turning Defense into Offense: Defensin Mimetics as Novel Antibiotics Targeting Lipid II

**DOI:** 10.1371/journal.ppat.1003732

**Published:** 2013-11-07

**Authors:** Kristen M. Varney, Alexandre M. J. J. Bonvin, Marzena Pazgier, Jakob Malin, Wenbo Yu, Eugene Ateh, Taiji Oashi, Wuyuan Lu, Jing Huang, Marlies Diepeveen-de Buin, Joseph Bryant, Eefjan Breukink, Alexander D. MacKerell, Erik P. H. de Leeuw

**Affiliations:** 1 NMR Facility, University of Maryland Baltimore School of Medicine, Baltimore, Maryland, United States of America; 2 Utrecht University, Bijvoet Center for Biomolecular Research, Faculty of Science-Chemistry, Utrecht, The Netherlands; 3 Institute of Human Virology & Department of Biochemistry and Molecular Biology of the University of Maryland Baltimore School of Medicine, Baltimore, Maryland, United States of America; 4 Maastricht University Medical Center, Maastricht, The Netherlands; 5 Department of Pharmaceutical Sciences and Computer-Aided Drug Design Center, University of Maryland, School of Pharmacy, Baltimore, Maryland, United States of America; Georgetown University, United States of America

## Abstract

We have previously reported on the functional interaction of Lipid II with human alpha-defensins, a class of antimicrobial peptides. Lipid II is an essential precursor for bacterial cell wall biosynthesis and an ideal and validated target for natural antibiotic compounds. Using a combination of structural, functional and *in silico* analyses, we present here the molecular basis for defensin-Lipid II binding. Based on the complex of Lipid II with Human Neutrophil peptide-1, we could identify and characterize chemically diverse low-molecular weight compounds that mimic the interactions between HNP-1 and Lipid II. Lead compound BAS00127538 was further characterized structurally and functionally; it specifically interacts with the N-acetyl muramic acid moiety and isoprenyl tail of Lipid II, targets cell wall synthesis and was protective in an *in vivo* model for sepsis. For the first time, we have identified and characterized low molecular weight synthetic compounds that target Lipid II with high specificity and affinity. Optimization of these compounds may allow for their development as novel, next generation therapeutic agents for the treatment of Gram-positive pathogenic infections.

## Author Summary

Every year, an increasing number of people are at risk for bacterial infections that cannot be effectively treated. This is because many bacteria are becoming more resistant to antibiotics. Of particular concern is the rise in hospital-acquired infections. Infection caused by the methicillin-resistant *Staphylococcus aureus* bacterium or MRSA is the cause of many fatalities and puts a burden on health care systems in many countries. The antibiotic of choice for treatment of *S. aureus* infections is vancomycin, an antimicrobial peptide that kills bacteria by binding to the bacterial cell wall component Lipid II. Here, we have identified for the first time, small synthetic compounds that also bind Lipid II with the aim to develop new antibiotic drugs to fight against bacterial infections.

## Introduction

The ever-increasing emergence of many pathogenic bacterial strains resistant to commonly used antibiotics is a rapidly growing concern in public health. Patients with weakened immunity because of chemotherapy, AIDS or organ transplantation or patients undergoing acute care in hospitals are significantly and increasingly at risk for acquiring opportunistic bacterial infections [1]. Seven leading groups of pathogens account for the increased risk for such infections, including four Gram-positive bacteria: *Staphylococcus aureus*, *Enterococcus faecium*, streptococci and coagulase-negative staphylococci [2]. Resistance against commonly used classical antibiotics has emerged in all of these pathogens.

The discovery and development of novel antibiotic compounds has been slow and our arsenal of effective antibiotics is dwindling. Resistant bacteria spread and cause infections at increasing rates, and thus there is an urgent need to develop novel classes of potent antibiotics against established molecular targets, such as Lipid II. Lipid II is an essential precursor in cell wall biosynthesis. It is comprised of a hydrophilic head group that includes a peptidoglycan subunit composed of N-acetylglucosamine (GlcNAc) and N-acetylmuramic acid (MurNAc) coupled to a short pentapeptide moiety. This headgroup is coupled to a long bactoprenol chain via a pyrophosphate group. The amount of Lipid II that can be synthesized is limited and the Lipid II molecule has a high turnover rate, making it an ideal and established molecular target for antibiotics [3], [4]. Four different classes of peptide antibiotics that target Lipid II have been described: (**1**) the glycopeptides, including vancomycin and teicoplanin; (**2**) the depsipeptide antibiotics, including ramoplanin and enduracidins; (**3**) the lantibiotics, including nisin and mersacidin and (**4**) cyclic peptides, including mannopeptimycins, plusbacin and katanosin B [5]–[11]. Recently, defensins were also found to target Lipid II. Defensins represent a major class of antimicrobial peptides found in almost all eukaryotes and prominently present in mammals [12]–[16]. Since our initial report on the functional interaction of the human defensin peptide HNP1 with Lipid II [17], several studies on defensins from other species has firmly established Lipid II as a target for these peptides. Most notably, Schneider *et al* [18] characterized the Lipid II binding site of the fungal defensin plectasin in molecular detail, putting defensins on the map as clinically relevant antimicrobial peptides. Two additional fungal defensins, oryzeacin (from *Aspergillus oryzae*) and eurocin (from *Eurotium amstelodami*) as well as two invertebrate defensins, lucifensin (from the blowfly *Lucilia sericata*) and gallicin (from the mussel *Mytilus galloprovinciali*), were shown to bind Lipid II in that study [18]. More recently, the spectrum of defensins binding Lipid II was widened further to include Human β-Defensin-3 [19] and three oyster defensins [20].

Strikingly, glycopeptides, defensins, depsipeptides, lantibiotics and cyclic peptides do not share any obvious sequence- or structural homology, yet all are able to specifically interact with Lipid II in the bacterial membrane environment. Here, we report on the unique interaction of HNP-1 with Lipid II. Based on molecular details of this interaction we further identify small compounds as defensin mimetics and report on their potential as novel antibiotic agents to fight against Gram-positive pathogens. The identified compounds represent the first non-natural, synthetic compounds that bind Lipid II and represent a novel class of molecules that have the potential to be developed into antibiotics that target Lipid II.

## Materials and Methods

### Materials

Chemicals used for solid phase peptide synthesis were obtained as described [21]. *Staphylococcus aureus* ATCC 29213 and *Escherichia coli* ATCC 25922 were obtained from Microbiologics (St. Cloud, MN). DiAcetyl-Lys-D-Alanine-D-Alanine (D-Ala), DiAcetyl-Lys-D-Alanine-D-Lac (D-Lac) and vancomycin were purchased from Sigma. Defensin mimetic compounds were obtained from various suppliers as listed in Table S1.

### Solid phase peptide synthesis

Chemical synthesis and folding of defensins was carried out as described [21], [22]. The molecular mass of the peptides was verified by electrospray ionization mass spectrometry (ESI-MS) as described [21]. Peptide stock solutions prepared with water were quantified spectroscopically using molar extinction coefficients at 280 nm calculated according to the algorithm of Pace et al [23].

### Lipid II purification

Lipid II was essentially generated as described [24]. Short-chain water-soluble Lipid II containing a lipid tail of three isoprene units (3-Lipid II or farnesyl-Lipid II) was generated and purified essentially as described [25].

### Surface Plasmon Resonance

Surface Plasmon Resonance binding experiments were carried out on a BIAcore T100 system (BIAcore Inc., Piscataway, NY) at 25°C. The assay buffer was 10 mM HEPES, 150 mM NaCl, 0.05% surfactant P20, pH 7.4 (±3 mM EDTA) supplemented with 10% DMSO. 3-Lipid II (50 RUs) was immobilized on CM5 sensor chips using the amine-coupling chemistry recommended by the manufacturer. For initial determination of binding, defensin mimetics were introduced into the flow-cells (30 µl/min) in the running buffer at 10 µM. Resonance signals were corrected for nonspecific binding by subtracting the background of the control flow-cell. After each analysis, the sensor chip surfaces were regenerated with 50 mM NaOH for 30 s at a flow rate 100 µl/min, and equilibrated with the buffer prior to next injection. For binding kinetics studies, binding isotherms were analyzed with manufacturer-supplied software for BIAcore T100.

### Antibacterial activity assay

The antibacterial activity of defensin mimetics against *Staphylococcus aureus* ATCC 29213 and *Escherichia coli* 25922 was carried out in a 96-well turbidimetric assay essentially as described previously [26] with the following modifications: bacteria were exposed for 30 min to compounds in 10 mM phosphate buffer containing 5% DMSO prior to addition of 2× Muller-Hinton medium. Bacterial growth was monitored for 12 hours and data were analyzed as described [26]. Determination of MICs was performed by Micromyx, LLC (Kalamazoo, Michigan) according to CLSI standards [27].

### Antagonization assays

Antagonization of the antibacterial activity of defensins against *Staphylococcus aureus* ATCC 29213 was carried out in a 96-well turbidimetric assay essentially as described previously [26]. Defensins (50 µM final concentration) were pre-incubated with 3-Lipid II at 1∶1, 1∶2.5 and 1∶5 defensin: Lipid II molar ratios for 30 min at RT. Following incubation, solutions were diluted two-fold in ten steps and bacteria were added. Defensin activity was neutralized by the addition of Mueller Hinton broth. Bacterial growth was monitored for 12 hours and data were analyzed as described [26].

### Crystallization and modeling of the HNP-1/Lipid II complex

Crystals were obtained using the hanging-drop vapor diffusion method at room temperature. Each drop contained 1 µl of HNP-1-Lipid II at ∼equimolar ratio and 1 µl of reservoir solution consisting of 0.2 M Sodium citrate tribasic dehydrate, 0.1 M HEPES sodium pH 7.5, 20% v/v 2-Propanol. Crystals grew typically in one week and were shaped as square plates of dimensions of approximately 0.2×0.2×0.1 mm. They belonged to the I432 space group, and each asymmetric unit contained 2 molecules of the complex. Data collection and refinement statistics are described in detail in **Table S1**.

The partial crystal structure of the complex, in which Lipid II could not be built entirely due to a lack of electron density, was subsequently used for generating a model of the complex by data-driven docking using the HADDOCK program (2.1 version) [28], [29]. The observed electron density around Ile20 of chain A, Leu25 of both chains and Arg15 of chain B was used to define ambiguous interaction restraints (AIRs) with an upper distance bound of 2 Å between the side chains of those residues and the soluble part of Lipid II (peptidic tail, oligosaccharide and pyrophosphate group). Random removal of restraints was turned off. One lipid II molecule was docked onto the HNP1 dimer with C2 symmetry restraints defined between the two HNP1 monomers. Topology and parameters for Lipid II were taken from [30]. Lipid II was treated as fully flexible during the refinement stage of HADDOCK. The docking was performed with default parameters, except for an increased number of models, 2000 at the rigid-body docking stage and 400 for subsequent flexible and explicit solvent refinement. The resulting models were clustered using a 7.5 Å RMSD cutoff and the clusters ranked based on the default HADDOCK score.

### Computer-Aided Drug Design (CADD)-database searching

Identification of Defensin mimetics involved two steps: 1) a 3D pharmacophore fingerprint typed atom triangles (TAT) [31] search and 2) a chemical/physiochemical similarity search with MACCS [32] and MPMFP [33] fingerprints performed using the program MOE (Chemical Computing Group Inc.) [31].

The first step was performed to find compounds that can mimic the chemical characteristic and relative spatial arrangement of the HNP-1 residue side chains that are important for binding with Lipid II. The full side chains of Ile20, Leu25 of monomer A and Arg15, Ile20 and Leu25 of monomer B from the experimentally solved complex structure were used as the reference for the pharmacophore search. As only the nitrogens of the Arg side chain serve as hydrogen-bond donors that interact with Lipid II, another reference structure with only the C-(NH_2_)_2_ moiety of the Arg15 side chain along with the full aliphatic side chains of other four key residues was also used for the pharmacophore search. To prepare compound databases for searching, 3D structures of low-molecular weight compounds were generated from 2D structures obtained from three large commercial databases; Maybridge (Thermo Fisher Scientific Inc., Wattham, MA), ChemBridge (San Diego, CA), and ChemDiv (San Diego, CA), which contain 59676, 482276, and 533143 compounds, respectively. The compounds were converted into 3D structures using MOE and subsequently minimized with the MMFF94 force field [34] to a root-mean-square (RMS) gradient of 0.05 kcal/mol/Å, followed by the assignment of 3D pharmacophore fingerprints for similarity searching. Pharmacophore searching was performed by comparing the small molecule 3D fingerprints with the HNP-1 dimer 3D pharmacophores with the extent of overlap calculated based on the Tanimoto similarity indices [35]. Database compounds with a Tanimoto index over selected cutoff values, with physiochemical properties that maximize bioavailability [36] and with unique chemical scaffolds were selected for the first round of biological experiments.

A second round of *in silico* searching was performed to find analogs of the five active compounds identified in the first round of pharmacophore searching and experimental testing. For each active compound, two individual similarity searches were performed to find compounds that are either structurally similar or physiochemically similar to the query compound, using MACCS or MPMFP fingerprints, respectively. An in-house database in the University of Maryland Computer-Aided Drug Design Center with 5.04 million compounds was used for searching. Database compounds with a Tanimoto index over selected cutoff values and with drug-like characteristics that maximize bioavailability [36] were selected for the second round of biological experiments.

### Macromolecular synthesis assays

Macromolecular synthesis inhibition by BAS00127538 and 1499-1221 were investigated using *S. aureus* MMX100 (ATCC 29213). Cells were grown at 35°C overnight on Tryptic Soy Agar Broth (Remel, Lenexa, KS), and growth from the plate was used to inoculate 15 ml of Mueller Hinton Broth. The culture was grown to early exponential growth phase (OD_600_ = 0.2 to 0.3) while incubating in a shaker at 35°C and 150 rpm. For each macromolecular assay, the test agents 1499-1221 and BAS00127538 were added at either 0, 0.25, 0.5, 1, 2, or 4, -fold their respective MIC values for *S. aureus* ATCC 29213. As positive control drugs, the following antibiotics were added at 8× MIC in order to validate each assay: Vancomycin (cell wall synthesis); ciprofloxacin (DNA synthesis), rifampin (RNA synthesis), cerulenin (lipid synthesis), and linezolid (protein synthesis).

For DNA and protein synthesis, 100 µl of cell culture reaching early exponential phase was added to triplicate wells containing various concentrations of test compound or control antibiotics (2.5 µl) at 40× the final concentration in 100% DMSO (0.1% methanol in water for Rifampicin). A 2.5% DMSO treated culture served as the “no drug” control for all experiments. Cells were added in 1.25× strength MHB to account for the volume of drug added to each reaction, or in M9 minimal medium for protein synthesis reactions. Following a 5 min incubation at room temperature either [^3^H]Thymidine (DNA synthesis) or [^3^H]Leucine (protein synthesis) was added at 0.5–1.0 µCi per reaction, depending on the experiment. Reactions were allowed to proceed at room temperature for 15–40 min and then stopped by adding 12 µl of cold 5% trichloroacetic acid (TCA) or 5% TCA/2% casamino acids (protein synthesis). Reactions were incubated on ice for 30 min and the TCA precipitated material was collected on a 25 mm GF/1.2 µm PES 96 well filter plate (Corning). After washing five times with 200 µl per well of cold 5% TCA, the filters were allowed to dry, and then counted using a Packard Top Count microplate scintillation counter.

For cell wall synthesis, bacterial cells in early exponential growth phase were transferred to M9 minimal medium and added to 1.5 ml eppendorf tubes (100 µl/tube) containing various concentrations of test compound or control antibiotics (2.5 µl) at 40× the final concentration in 100% DMSO as described above. Following a 5 min incubation at 37°C, [^14^C] N-acetyl-glucosamine (0.4 µCi/reaction) was added to each tube and incubated for 45 min in a 37°C heating block. Reactions were stopped through the addition of 100 µl of 8% SDS to each tube. Reactions were then heated at 95°C for 30 min in a heating block, cooled, briefly centrifuged, and spotted onto pre-wet HA filters (0.45 µM). After washing three times with 5 ml of 0.1% SDS, the filters were rinsed two times with 5 ml of deionized water, allowed to dry, and then counted using a Beckman LS3801 liquid scintillation counter.

For lipid synthesis, bacterial cells were grown to early exponential growth phase in MHB and 100 µl was added to 1.5 ml Eppendorf tubes (in triplicate) containing various concentrations of test compound or control antibiotics as described above. Following a 5 min incubation at RT, [^3^H] glycerol was added at 0.5 µCi per reaction. Reactions were allowed to proceed at room temperature for 40 min and then stopped through the addition of 375 µl of chloroform/methanol (1∶2) followed by vortexing for 20 sec after. Chloroform (125 µl) was then added to each reaction and vortexed, followed by the addition of 125 µl dH_2_O and vortexing. Reactions were centrifuged at 13,000 rpm for 10 min, and then 150 µl of the organic phase was transferred to a scintillation vial and allowed to dry in a fume hood for at least 1 hr. Samples were then counted via liquid scintillation counting. Analyses were performed by Micromyx, LLC (Kalamazoo, Michigan).

### LUV leakage assay

Large unilamellar vesicles were prepared by the extrusion technique [37]. Vesicles were made of Di-Palmoyl-Phosphatidyl Choline (DPPC, Avanti Polar Lipids) with or without 0.1 mol % Lipid II. Vesicles were prepared with 50 mM rhodamine, washed with saline solution and purified by G25 Sephadex column (GE healthcare). Compounds were diluted in saline solution in 96-well plate format and vesicles were added to each well. The increase of fluorescence intensity was measured at 612 nm (excitation at 544 nm) on a Molecular Dynamics spectrophotometer at 20 °C. Compound-induced leakage was expressed relative to the total amount of rhodamine released after lysis of the vesicles by addition of 10 µl of 20% Triton X-100.

### Red Blood Cell (RBC) lysis

The method described by Stasiuk et al. [38] was followed in general. Defibrinated human blood (Valley BioMedical) was washed three times with buffer (10 mM Tris-HCl, pH 7.4, 0.9% NaCl) and resuspended to a final concentration of 3% RBCs immediately prior to performing the assay. One hundred eighty microliters of RBCs were added to 1020 µl buffer containing various concentrations of the investigational compound. A total of 4 different compound concentrations (based upon the broth dilution MIC) were tested in duplicate at the following multiples of the MIC values: 0, 1× MIC, 4× MIC, 8× MIC, and 16× MIC. DMSO alone (5% final concentration) served as the negative control to subtract the background, while a reaction with water substituted for buffer served as a positive control that completely lyses the RBCs. Vancomycin served as assay validation (negative control) and was used 0, 1× MIC, 4× MIC, 8× MIC, and 16× MIC also. Incubation of compounds and RBCs proceeded for 30 minutes at room temperature, followed by centrifugation at 1,300× *g* for 5 minutes to pellet the RBCs. Finally, 300 µl of the supernatant was removed to a 96-well plate and the released hemoglobin was measured at A540 using a SpectraMax (Molecular Dynamics) plate reader. Prism (GraphPad) software was used for data analysis. Results were expressed as percent lysis compared to treatment of RBCs with deionized water, which completely ruptures the membrane. Analyses were performed by Micromyx, LLC (Kalamazoo, Michigan).

### Nuclear Magnetic Resonance

The NMR samples consisted of 0.15 mM Lipid II, 0.15 mM BAS00127538 compound, or 0.15 mM Lipid II+0.15 mM BAS00127538 compound. All samples were dissolved in 90% double distilled H_2_0+10% DMSO, incubated for 30 minutes, freeze-dried, and then dissolved in 300 µL of d6-DMSO. NMR experiments were carried out at 25°C on an 800 MHz Bruker Avance NMR spectrometer (800.27 MHz for protons) equipped with a pulse-field gradient unit, four frequency channels, and a triple resonance TXI cryoprobe (Bruker Biospin, Billerica, MA). 1D proton experiments were collected to probe for chemical shift changes and 2D TOCSY (30, 60, and 90 msec spinlock times), 2D NOESY (150 and 300 msec mixing times), and natural abundance ^13^C-HSQC experiments were collected to determine proton and carbon chemical shift assignments.

### Molecular modeling of the BAS00127538-Lipid II complex

A model of the BAS00127538-Lipid II complex was generated based on the experimental data followed by molecular dynamics (MD) simulations. Lipid II, which consists of a pentapeptide (L-Ala-D-γ-Glu-L-Lys-D-Ala-D-Ala), two cyclic sugars, N-acetylglucosamine (GlcNAc) and N-acetylmuramic acid (MurNAc), and a di-phosphate prenyl chain was generated in the program CHARMM [39] using the additive CHARMM force field for proteins and carbohydrates [40]–[43]. This involved creation of new topology files for MurNac, D-γ-Glu and the di-phosphate prenyl chain with missing parameters assigned by analogy. BAS00127538 was generated with the CHARMM general force field (CGenFF) [44]. The starting conformation of Lipid II was obtained from the experimental NMR structure of the nisin-Lipid II complex (pdb code: 1WCO) [30] followed by a 2000 step steepest descent (SD) minimization and then a 200 step adopted basis Newton-Raphson (ABNR) minimization yielding a conformation with a root-mean-square (RMS) difference of 4.7 Å for all non-hydrogen atoms as compared with the experimental NMR structure. The inhibitor-Lipid II model was built by orienting the inhibitor adjacent to Lipid II based on data from the NMR experiments. This involved manually placing one of the inhibitor benzene rings and MurNac ring in Lipid II adjacent to each other. Harmonic restraints, k(r-r_0_)^2^, were placed between the geometric centers of the above groups, where k = 50 kcal/(mol Å^2^), r_0_ = 3 Å and r is the distance between those geometric centers. The system was then subjected to a 2000 step SD energy minimization followed by a 1 ns gas phase Langevin simulation in the presence of the restraints followed by an additional 1 ns gas phase Langevin simulation in the absence of the restraints. The resulting complex was then solvated in a 48*48*48 Å^3^ pre-equilibrated [45] water box for condensed phase simulations. All water molecules within 2.8 Å of the non-hydrogen atoms of the complex are removed, and two sodium ions were added to neutralize the system, which contained 10385 atoms. While all nonbonded interactions were evaluated for gas phase simulations, nonbonded interactions were truncated at 12 Å for condensed phase simulations, with a force switch smoothing from 10 to 12 Å. Simulations were performed using periodic boundary conditions with the particle mesh Ewald summation method [46] used to treat the electrostatic interactions with a real space cutoff of 12 Å. The system was minimized for 2000 SD steps and subjected to an isobaric, isothermal (NPT) MD simulation at 300 K and 1 atm. Simulations were extended for 2 ns during which the inhibitor remains in close contact with Lipid II.

### Murine peritoneal sepsis model

#### Ethics statement

Care of the mice met or exceeded the standards set forth by the National Institute of Health Guide for the care and use of laboratory animals and the AVMA panel on Euthanasia. All procedures in this study have been approved by the Institutional Animal Care and Use Committee (IACUC) at the University of Maryland Baltimore School of Medicine (Protocol number 02122005). Adult C57BL/6J mice (∼18 grams, 8–10 weeks old) were used for all experiments. Mice were obtained from the Jackson Laboratory (Bar Harbor, Maine, USA) and housed in the IHV SPC animal core facility. Mice were fed standard chow (Harlan Laboratories) and water ad libitum. To assess the protective potency of defensin mimetic BAS00127538, groups of 5 mice were inoculated intraperitoneally ∼10^7^ CFU/mL of *S. aureus* ATCC 29213 in 500 µL saline solution/25% DMSO plus 4.5% (w/v) porcine gastric mucin (Sigma Chemical Co., St. Louis, MO). Infected animals (*n = 5*) were subsequently treated by intra-peritoneal injection 1 and 4 hours post-infection with 2.5 mg/kg of compound in 100 µL sterile saline solution plus 25% DMSO (V/V), vancomycin/lysostaphin (5 mg/kg, saline solution/25% DMSO) or vehicle (saline solution/25% DMSO) as positive and negative controls, respectively. Animals were closely observed during a period of 24 hours and mice that show signs of severe sepsis were humanely euthanized. Blood samples were collected by retro-orbital puncture at the indicated intervals post-infection using lithium-heparin polystyrene tubes to prevent coagulation. Spleens were harvested aseptically, weighed and homogenized in 500 µl of sterile saline solution using an IKA T10 basic disperser (IKA, Wilmington NC). Whole blood samples and spleen homogenates were serially diluted and plated onto LB agar plates. Bacterial counts were determined following 24 h incubation at 37°C and expressed as CFU per milliliter for blood and CFU per gram for spleen.

## Results

### HNP-1 in complex with Lipid II

We have previously determined the crystal structure of chemically synthesized, wild-type HNP-1 at 1.6 Å resolution [47]. We attempted co-crystallization of a HNP-1/Lipid II complex. HNP-1 and soluble 3-Lipid II were mixed in a 1∶1 molar ratio. Crystals were observed in three separate crystallization conditions and all belonged to the same space group. Importantly, both crystallization conditions and space group were different from those for HNP1 alone. Models based on the monomer of HNP-1 (PDB:1GNY, [48]) and lipid II (PDB: 1WCO, [49]) as a probes were initially used in molecular replacement experiments to define a structure of the complex from the crystals growing from the solution containing HNP-1-Lipid II complex. Matthews coefficient analysis of protein crystal solvent content of I432 crystals indicated three molecules of HNP-1 monomer or HNP-1-Lipid II complex composed of two HNP-1 molecules and one lipid molecule in the crystallographic asymmetric unit. With Phaser we were unambiguously able to define two HNP-1, but not three HNP-1 molecules which were arranged into wild-type HNP-1 dimer. Calculated 2*F*_o_ − *F*_c_ electron density maps for this model clearly indicated the presence of additional density in proximity of the R15, Ile20 and Leu25 side chains (**Figure S1**). Pairwise superimposition analysis of HNP-1 alone or HNP-1 in complex with Lipid II revealed very close similarity as shown by an average RMSD value of 0.8 Å for 60 aligned Cα atoms. Although the overall structure of the dimers is the same, their pairwise superimposition indicates an apparent shift of the monomer B backbone forming β1/β2 and β2/β3 connecting loops and the β3 strand (**Figure S2**). In the dimer of crystals grown from HNP1-lipid II mixture the backbone atom of L_a_25 and R_a_15 identified by HADDOCK to be involved in Lipid II interaction (see below) do show positional shifts of around 2.0 and 1.3 Å, respectively (**Figure S3**).

**Figure 1:**
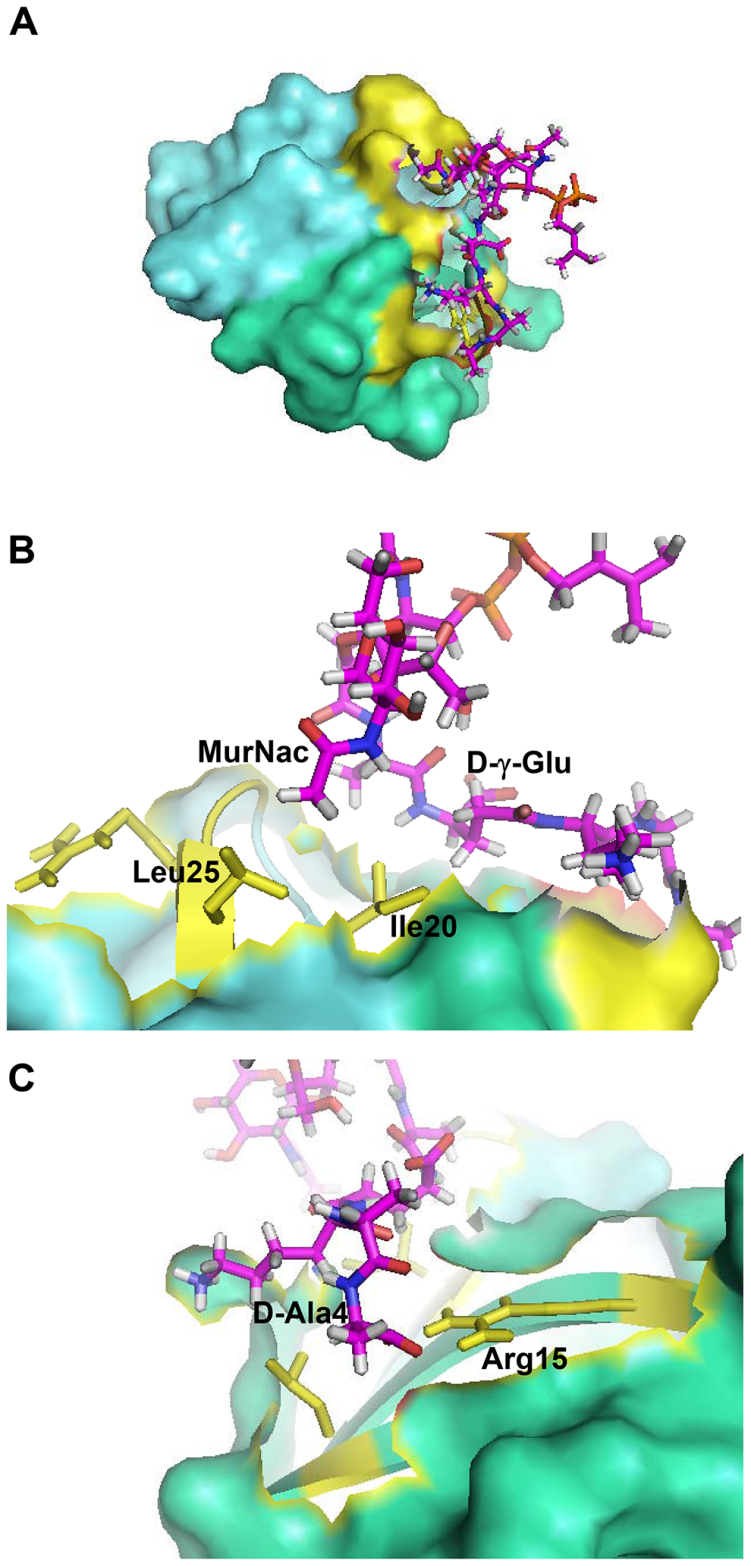
Crystal structure-based model of the HNP-1-Lipid II complex obtained with HADDOCK. (A) The HNP-1 dimer is shown in surface representation. (**B**) Residue Isoleucine 20 of HNP-1 monomer A interacts with Lysine-3 of the Lipid II pentapeptide, whereas Leucine residue 25 of HNP-1 monomer A interacts with D-Ala at position 4. (**C**) Residues Arginine15, Isoleucine 20 and Leucine25 of HNP-1 monomer B interact with γD-Glu-2 and the phosphate/N-acetyl muramic acid moiety of Lipid II. Critical residues in HNP-1 for the Lipid II interactions are shown in yellow and span the two monomers indicated in green and blue.

To visualize the complex between HNP1 and Lipid II, X-ray directed docking studies using the HADDOCK program [28] were performed. Our partial complex crystal structure, together with the availability of the HNP-1 and Lipid II experimental 3D structures, made such modeling feasible. Based on the X-ray data, the amino acid side-chains of Ile20 and Leu25 of monomer A and R15, Ile20 and Leu25 of monomer B of HNP1 form the primary Lipid II binding site of HNP1 and this information was used to drive the docking (see Material and Methods). Two clusters of solutions were obtained (**Table S2**). A view of the top ranking solution from the best scoring and most populated cluster is shown in **Figure 1** and contact residues are listed in **Table 1**. The interaction between HNP-1 and Lipid II involves mainly van der Waals (vDW) interactions and one main chain-side chain hydrogen bond between Arg15 of HNP-1 Monomer B and D-Ala at position four of the Lipid-II pentapeptide. Ile20 of Monomer A forms vDW interactions with three residues of the Lipid II pentapeptide as well as the N-acetyl muramic acid (NAM) moiety. The leucines at positions 25 of both monomers interact with the NAM moiety as well. Residues Gly23 and Arg24 of the HNP-1 A monomer are involved in additional interactions.

**Table 1:** Residues involved in HNP-1 Lipid II contacts.

3-Lipid II	HNP-1	Nature of interaction
**MurNac**	Ile20, Arg24, Leu25 (monomer A), Leu25 (Monomer B)	van der Waals (4)
**Ala1**	Ile20, Gly23 (Monomer A)	van der Waals (2)
**Lys3**	Ile20 (Monomer A)	van der Waals (1)
**D-Ala4**	Arg15 (Monomer B)	Predicted Hydrogen bonding (1)

Common three letters abbreviation is used for amino acids. D: amino acid in D-configuration. MurNac: N-acetyl Muramic acid. The identified contacts are based on an analysis of the top 4 docking models.

**Figure 2:**
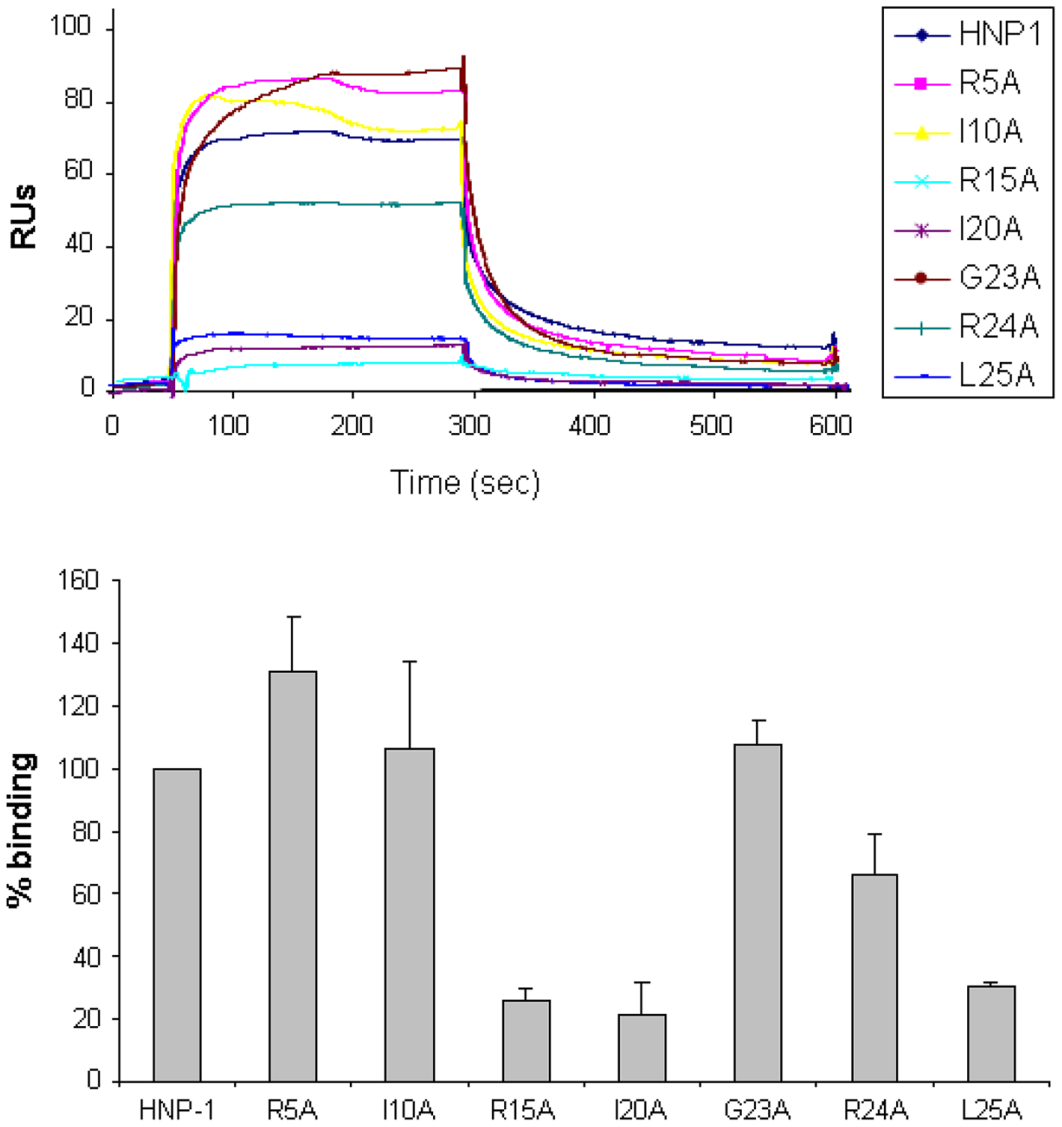
Binding of HNP-1 and HNP-1 single alanine mutants on immobilized Lipid II as determined by SPR. (Upper panel) Representative sensorgrams of one out of two separate experiments of HNP-1 and analogues at 10 µM using a sensorchip with 40 RUs of soluble, 3-Lipid II. (Lower panel) Quantification of binding of HNP-1 mutants compared to binding of wild-type HNP-1, set as 100%.

**Figure 3:**
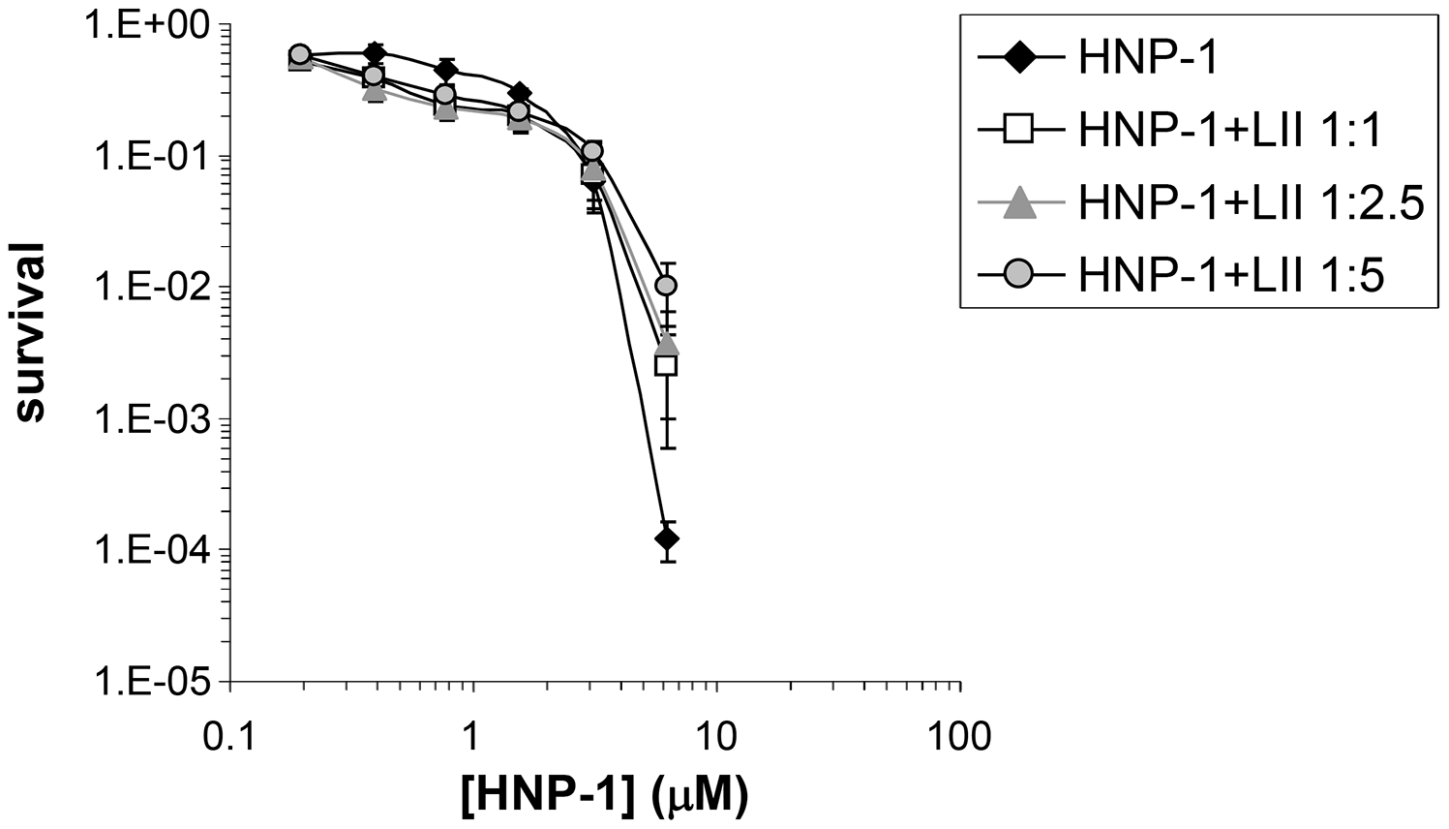
Lipid II antagonizes the antibacterial activity of HNP-1. Survival curves of *S. aureus* ATCC 29213 exposed to HNP-1 (at concentrations varying two-fold from 50 to 1.25 µM). Defensin peptide was pre-incubated with 3-Lipid II at varying molar ratios for 30 min prior to addition of bacteria. Bacteria were subsequently exposed to HNP-1 for 30 min. Each curve is the mean of three separate experiments (±S.D.). Points scored as zero survival could not be plotted.

Since our docking model predicts Arg15, Ile20, Gly23, Arg24 and Leu25 to form the Lipid II binding site, we would expect that replacement of these residues by alanine will affect Lipid II binding and bacterial killing directly. We therefore assayed for Lipid II binding directly by Surface Plasmon Resonance using single alanine mutants of HNP-1 [50]. As expected, replacement of the most critical residues forming the predicted Lipid II binding site by alanine (Arg15, Ile20 and Leu25) resulted in significant reduction of binding to Lipid II as compared to the wild-type HNP-1 (**Figure 2**). In contrast, replacement of Arg5, Ile10 or Gly23 by alanine did not affect binding to Lipid II, indicating that these residues are not important for Lipid II binding. The HNP-1 R24A mutant maintained significant binding to Lipid II, suggesting that this residue contributes, but does not make a critical contribution to Lipid II binding. Next, we examined whether the antibacterial activity of HNP-1 could be antagonized by soluble Lipid II as a measure for functional interaction. HNP-1 (50 µM) was pre-incubated with 3-Lipid II at varying molar ratios and killing of *S. aureus* was determined using the vCC protocol [26] (**Figure 3**). The bactericidal activity of the HNP-1 peptide appeared partly antagonized by one order of magnitude by the presence of Lipid II in a 1∶1 molar ratio (one order of magnitude), suggesting additional mechanism are involved in bacterial killing for this defensin.

### Identification and classification of defensin mimetics

Given the antimicrobial activity of HNP-1, we reasoned that compounds that mimic the interaction between HNP-1 and Lipid II could have potential antibiotic use. To identify low molecular weight compounds that can mimic the spatial orientation of the side chains in the HNP-1 dimer that bind Lipid II, a search of commercially available drug-like compounds was undertaken. 3D TAT pharmacophore fingerprints were used to describe the physical properties and spatial relationships of residues Ile20 and Leu25 of monomer A, and Arg15, Ile20 and Leu25 of monomer B in the HNP-1 dimer. This information was then used in a pharmacophore search to identify compounds with the desired features. After the first round of biological testing, five active compounds were identified and two types of similarity searching were conducted. The first method is based on chemical similarity and may potentially identify compounds with improved activity as well as produce data allowing for a structure-activity relationship for the compounds to be developed that may be of utility of subsequent ligand design. Searching was also performed based on physiochemical properties that may lead to the identification of novel chemical structures that represent new lead compounds [51]. In total, 75 compounds from the two rounds of similarity searches were selected. All compounds were tested for antibacterial activity, binding to Lipid II by Surface Plasmon Resonance and for cytotoxicity against two human cell lines. Out of 75 compounds, 28 (37.6%) were identified that showed specific killing against *S. aureus* over *E. coli*. Seventeen compounds (22.6%) showed significant binding to Lipid II. 6.6% of all compounds were equally active against *S. aureus* and *E. coli* (5/75) and 46% (42/75) showed no activity (42/75). Results for all compounds are summarized in **Table S3**.

### Functional characterization of lead defensin mimetics

**Figure 4:**
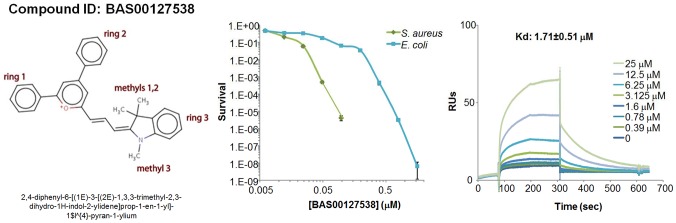
Characterization of BAS00127538. Chemical structure (left panel), bacterial killing (middle panel) and Lipid II binding (right panel) of defensin mimetic BAS00127538. Mimetic compound was 100% bactericidal at 0.244 µM against *S. aureus* and 7.8 µM against *E. coli*. Points of zero survival could not be plotted. (right panel) Representative sensorgrams of one out of three experiments of BAS00127538 binding to immobilized 3-Lipid II.

Based on the assays described in Supplementary Table S3, the low-molecular weight compounds selected as potential defensin mimetics were classified based on chemical structures, Lipid II binding, cytotoxicity and preferential Gram-positive killing (**Table 2**). **Figure 4** shows the results for lead compound BAS00127538 as an example. This compound most strongly bound to Lipid II as measured by SPR and potently killed *S. aureus* bacteria. To confirm the antibacterial killing assays, Minimal Inhibitory Concentrations (MICs, µg/ml) were determined for lead compounds against clinically relevant bacterial strains (**Table 3**). In agreement with the killing assays, lead defensin mimetics tested were potently active against Gram-positive isolates, and generally no activity was apparent against Gram-negative isolates, with the exception of BAS00127538, which had MICs of 4 µg/ml when tested against *E. coli*. There was no significant difference for any compound when evaluated against clinically relevant strains (e.g. MRSA, VRE, PRSP).

**Table 2:** Classification of lead defensin mimetics.

Compound ID	Bacterial Killing (µM)		Lipid II binding (*K*_d_, µM)	Cytotoxicity (C_50%_, µM)	
	*S. aureus*	*E. coli*		*Caco-2*	*Jurkat*
**Class I**					
BAS00127538	0.244	7.8	1.71	2.7	14.5
1499-1221	0.031	125	N.D.	8.9	18.6
1493-0289	3.9	125	4.26	6.8	41.7
**Class II**					
5107930	23.4	125	3.64	27.9	>223
4090-1978	15.6	62.5	N.D.	16.7	87.3
**Class III**					
363003	3.9	>500	39.5	3.5	12.7
1611-0203	3.9	>500	59.6	4.2	63.4

N.D: Not Determinable by SPR. Bacterial killing: Concentration resulting in 100% killing after exposure of compound to bacteria for 30 min, determined by modified vCC assays [26]. Binding to immobilized 3-Lipid II was analyzed by Surface Plasmon Resonance. C_50%_ equals compound concentration resulting in 50% cell survival measured by MTT assay following incubation for 24 h (Caco-2) or 4 h (Jurkat).

**Table 3:** Broth microdilution susceptibility testing for lead defensin mimetics and comparators.

Organism:	MMX#-ATCC#	BAS00127538	1499-1221	1493-0289	5109730	4090-1978	363003	1611-0203	Ciprofloxacin	Linezolid
***Staphylococcus aureus***	**100-29213**	0.5	0.25	1	16	2	1	2	0.5	4
***Staphylococcus aureus*** ** (MRSA)**	**757-NA**	0.5	0.5	2	16	2	1	2	>2	4
***Enterococcus faecalis***	**101-29212**	1	2	2	16	16	1	1	1	2
***Enterococcus faecalis*** ** (VRE)**	**848-NA**	1	2	4	16	16	1	1	>2	2
***Streptococcus pneumoniae***	**1195-49619**	8	>8	16	>64	>8	8	>16	1	2
***Streptococcus pneumonia*** ** (PRSP)**	**884-NA**	8	>8	16	>64	>8	16	>16	2	2
***Escherichia coli***	**102-25922**	4	>16	>8	>16	64	>32	>4	0.008	>64
***Pseudomonas aeruginosa***	**103-27853**	>8	>16	>8	>16	>64	>32	>4	0.25	>64

Reported MICs (µg/mL) were adjusted to reflect instances where drug precipitation obscured the interpretation of the endpoint.

NA-not applicable, MRSA-methicillin-resistant *S. aureus*, VRE-vancomycin-resistant enterococci, PRSP-penicillin-resistant *S. Pneumoniae*. Experiments were carried out according to CLSI standards [27] by Micromyx, LLC (Kalamazoo, Michigan). MIC values for comparators (Ciprofloxin and Linezolid) were within QC range [62].

**Figure 5:**
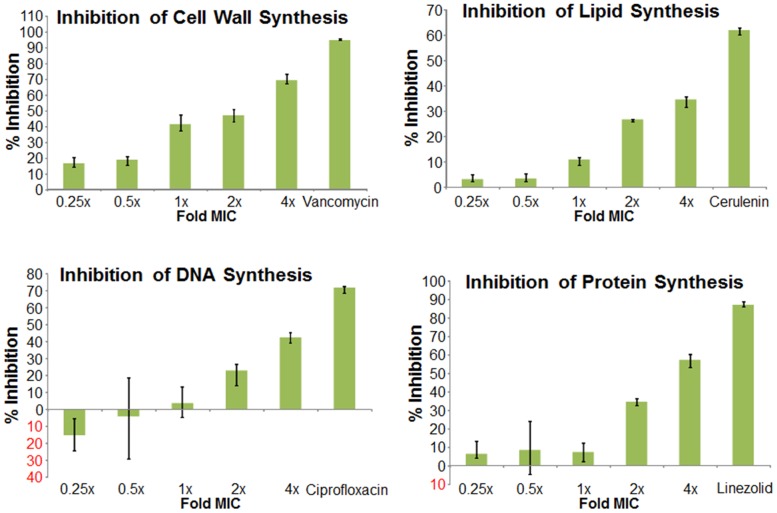
Mechanism of action studies of BAS00127538. Exponentially growing *S. aureus 29213* cells were exposed to compound and comparators in triplicate using 2.5% DMSO as “no drug” control. Cells were added to Mueller-Hinton Broth or M9 medium for protein synthesis and further incubated in the presence of [^14^C]N-acetyl glucosamine (cell wall), [^3^H]glycerol (lipid), [^3^H]Thymidine (DNA), or [^3^H]Leucine (protein). Following incubation, reactions were stopped by adding TCA (DNA, protein), 8% SDS (cell wall) or chloroform/methanol (lipid) and analyzed by scintillation counting.

**Figure 6:**
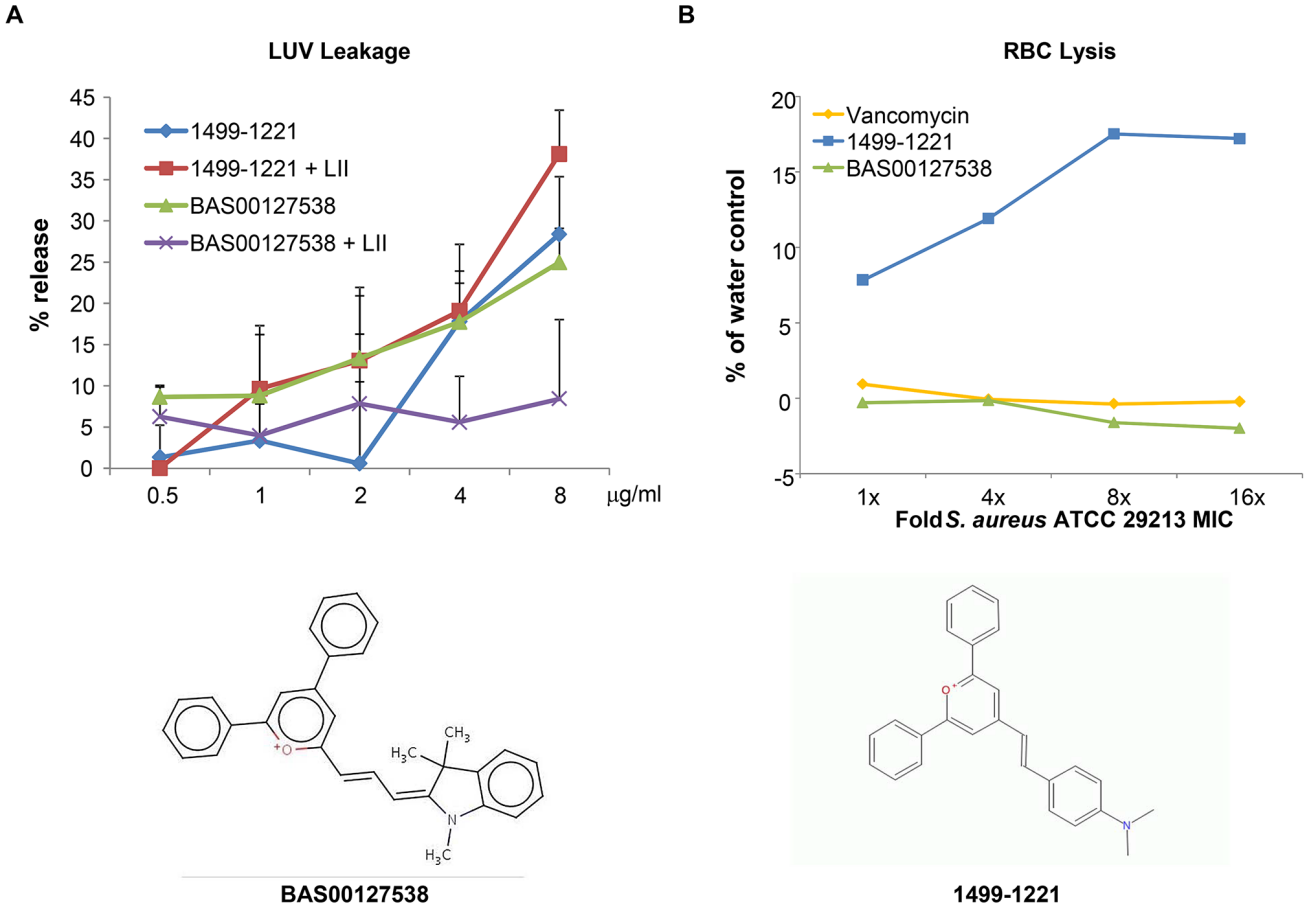
Membrane activity of BAS00127538 and 1499-1221. (**A**) Compounds were diluted in sextuplet in phosphate-buffered saline solution and incubated for 30 min at RT with DPPC LUVs with or without Lipid II as indicated. Release of fluorophore was expressed as percentage of release compared to controls containing Triton X-100. Results represent average of three experiments, each carried out in sextuplet (**B**) Defibrinated human blood was washed three times with buffer (10 mM Tris-HCl, pH 7.4, 0.9% NaCl) and was resuspended to a final concentration of 3% RBCs immediately prior to performing the assay. Incubation of compounds and RBCs was for 30 minutes at room temperature, followed by centrifugation at 1,300× *g* for 5 minutes to pellet the RBCs. 300 µl of the supernatant was removed to a 96-well plate and the released hemoglobin was measured at A540 using a SpectraMax (Molecular Dynamics) plate reader.

We next used mechanism of action studies to determine the mode of bacterial killing by BAS00127538 (**Figure 5**). At 1× MIC, BAS00127538 significantly inhibited cell wall synthesis, but not DNA, lipid or protein synthesis, indicating that cell wall synthesis is the primary target. At elevated concentrations, DNA, protein and lipid synthesis were reduced also, suggesting that the compound acts through a secondary mechanism, the most likely of which is membrane perturbation. We therefore determined membrane perturbation of BAS00127538 by examining its ability to cause lysis of Large Unilamellar Vesicles (LUVs) or red blood cells (RBCs). We find that BAS00127538 induces significant leakage of LUVs at 8 µg/ml (equates to 16× MIC for *S. aureus*), but does not induce lysis of red blood cells (**Figure 6**). Further, we find that lysis of LUVs induced by BAS00127538 is reduced by the presence of Lipid II. We also used a second mimetic compound, 1499-1221, in these studies. This compound is structurally related to BAS00127538 (see **Figure 6**) and potently kills Gram-positive organisms, however, Lipid II binding by NMR could not be confirmed for this compound using the approaches that were successful for BAS00127538. Compound 1499-1221 induced significant membrane rupture in LUVs irrespective of Lipid II as well as RBCs. Mechanism of action studies for this compound indicate that membrane perturbation is the likely primary mechanism for this compound (**Figure S4**).

### Interaction of lead defensin mimetic BAS00127538 with Lipid II

**Figure 7:**
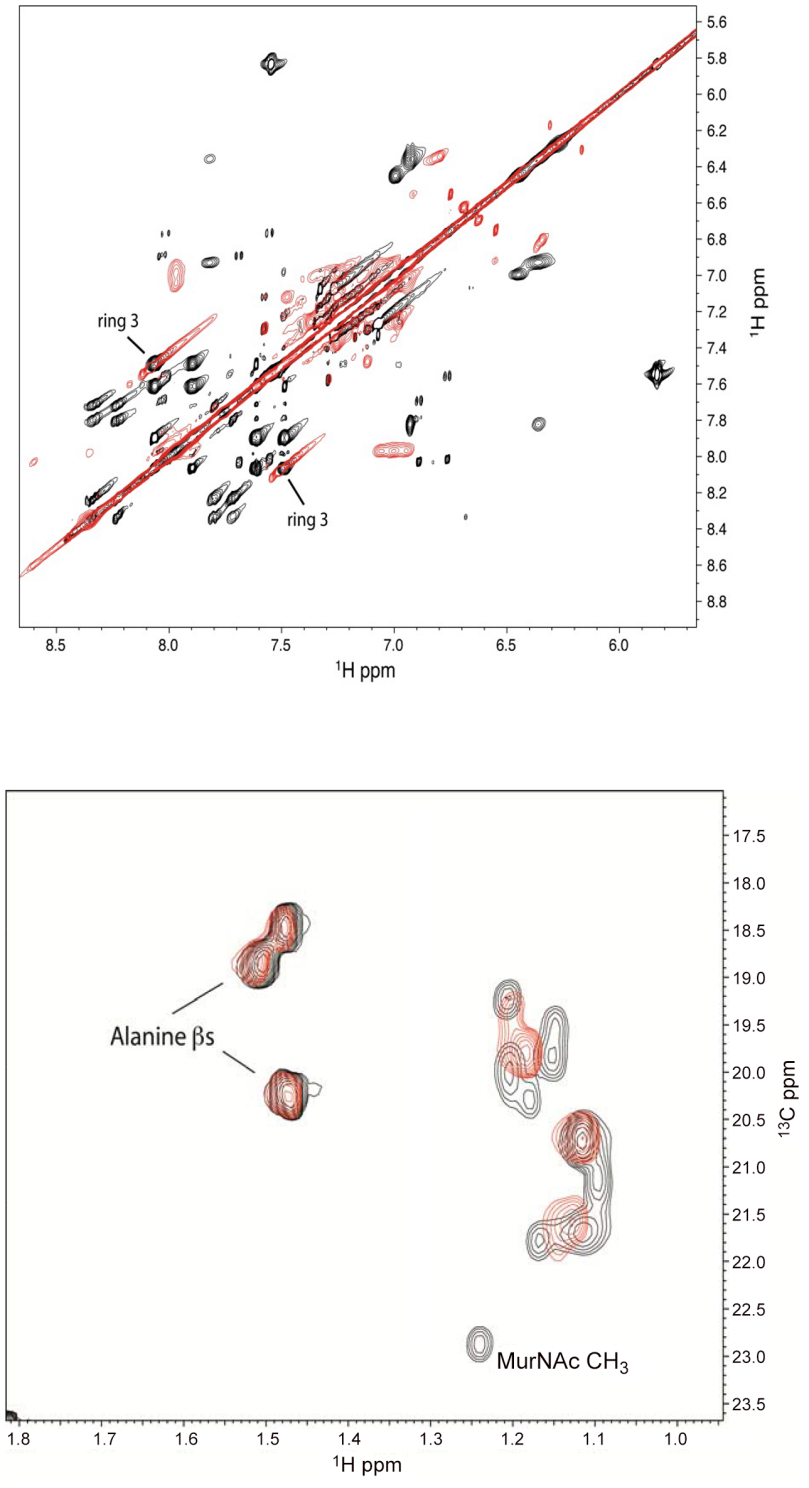
Analysis of the BAS00127538-lipid II complex by NMR. (Upper panel) Analysis of 2D TOCSY spectra collected at 800 MHz of the aromatic region of compound BAS00127538 alone (black) overlaid with spectra of compound bound to Lipid II (red) (Lower panel). 2D natural abunance ^13^C HSQC spectrum illustrating the interaction between Lipid II and the compound BAS00127538. BAS00127538 alone (black) is overlaid with a spectrum of compound bound to Lipid II (red). Spectra were collected on a Bruker 800 MHz Avance NMR spectrometer at 25 degrees. Chemical shift changes for Lipid II upon BAS00127538 compound binding suggest that the interaction is occuring at or near the MurNAc moeity of Lipid II.

To confirm the binding of defensins mimetic BAS00127538 to Lipid II we observed by SPR, their interaction was studied directly by NMR (Figure 7). Specifically, we used 1D proton NMR spectra to determine if any chemical shift changes occur when the compound was added to 3-Lipid II. BAS00127538 was found to interact and was analyzed further by 2D TOCSY, NOESY, and natural abundance ^13^C HSQC analyses (Figure 7, upper panel). Large chemical shifts were observed on the face of this compound that contains two aromatic rings (Figure 5). No chemical shifts were observed for the methyl groups on the opposite side of the molecule (not shown). Analysis of 3-Lipid II NMR spectra with and without compound allowed the interaction to be pinpointed to the N-Acetylmuramic acid moiety (MurNAc) of lipid II (Figure 7, lower panel). No chemical shifts for the pentapeptide Alanine residues were observed.

**Figure 8:**
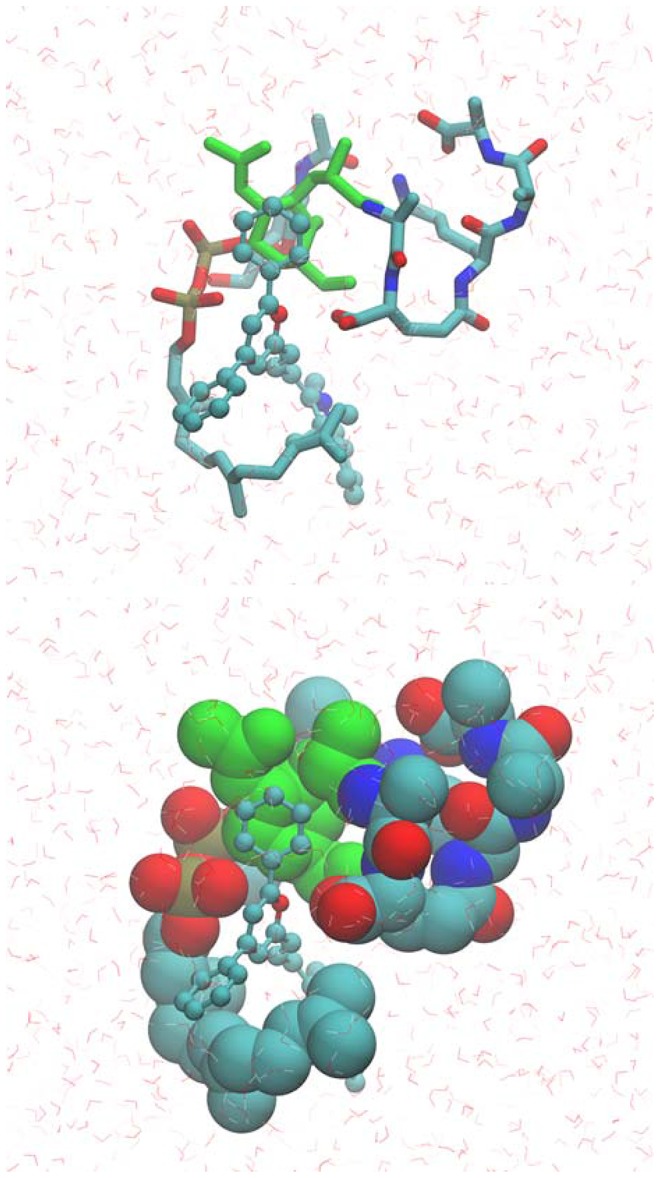
Model of the BAS00127538-lipid II complex obtained with CADD in conjunction with the NMR data. Upper panel includes BAS00127538 shown in CPK atom-colored representation, Lipid II in a licorice, atom-colored representation, with the exception of the N-MurNac moeity, which is green, and water molecules included in the simulation are shown in stick format. Lower panel is the same as upper panel except Lipid II is shown in van der Waals representation. Images created with VMD [63].

Based on the NMR data, MD simulations were used to model the BAS00127538-3-Lipid II complex. This involved initially restraining each aromatic ring to be adjacent to MurNAc followed by explicit solvent MD simulations in which the restraint was removed following an equilibration period. The resulting model, which was stable in the explicit solvent MD simulation, is shown in **Figure 8**. One aromatic ring of BAS00127538 lies over the MurNAc moiety (green) of Lipid II (bond, atom color except for MurNAc in green) consistent with the NMR data with the positively charged pyran ring of the inhibitor between the phosphate and acid moieties of Lipid II. In addition, the isoprenyl tail of lipid II forms a hydrophobic pocket that interacts with the second aromatic ring and the linker to indolylene ring.

### *In vivo* efficacy of BAS00127538

**Figure 9:**
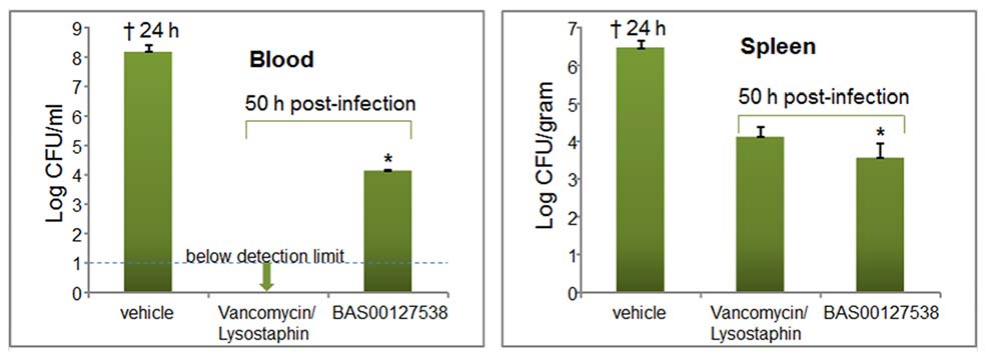
Efficacy of BAS00127538 *in vivo*. Blood samples were collected from vehicle-treated animals at 20 h or at 50 h post-infection from vancomycin and BAS00127538-treated animals. * One animal treated with compound did not survive beyond 28 h.

We established a murine model for sepsis to evaluate the efficacy of our lead defensin mimetics as antibiotic agents *in vivo*. Preliminary experiments indicated that the lead compounds listed in **Table 2** were effective at 5 mg/kg in clearing non-lethal doses of *S. aureus* 29213 bacteria when administered intraperitoneally (not shown). Lead compound BAS00127538 proved most efficacious and was selected for further experimentation. Mice (n = 5) were inoculated intraperitoneally with *S. aureus* 29213 and treated 1 h and 4 h post-infection with compound BAS00127538 at 2.5 mg/kg intraperitoneally. Animals were monitored for survival and blood and spleen samples were collected. Bacterial counts were determined and compared to control treatment with vancomycin/lysostaphin as measures of efficacy (**Figure 9**). Animals treated with vehicle did not survive after 24 h. Animals treated with vancomycin/lysostaphin survived the length of the experiment and bacterial counts in blood and spleen were in accordance with published data [52]. Treatment with BAS00127538 resulted in survival of 4 out of five animals and significantly reduced bacterial counts in spleen and blood, indicative of *in vivo* antibiotic efficacy.

## Discussion

The view on how antimicrobial peptides kill micro-organisms has been nuanced in the last few years. The broad traditional view of killing comprises an initial phase of electrostatic attraction of mostly cationic peptides to negatively charged molecules on the surface of micro-organisms [53]. Following the initial interaction, antimicrobial peptides disrupt membrane integrity, causing leakage of cellular content and cell death. In fact, synthetic compounds that cause membrane disruption are effective antimicrobials and have been extensively studied [54], [55]. Their killing mechanism depends on a distribution of positive charge and hydrophobicity, is largely species-independent and does not involve a specific bacterial target molecule. A functional interaction between defensins and Lipid II has only recently been described as a novel way by which these versatile peptides act against bacteria [17], [18]. In their landmark report, Schneider *et al* reported on the fungal defensin plectasin binding to Lipid II. The study identified interactions between plectasin and the solvent-exposed pyrophosphate region of Lipid II [18]. These interactions involved residues Phe2, Glu3, Cys4 and C27 as well as the N-terminus and His18 side chain of this defensin. Importantly, the binding sites of plectasin to Lipid II do not overlap with the vancomycin binding site on Lipid II. The mechanism of resistance to vancomycin involves specific modifications of the amino acid composition of the pentapeptide in the Lipid II molecule [56]. Such modifications often occur rapidly within bacterial populations, likely due to the high degree of flexibility and variability of amino acid synthesis and incorporation [56].

Lipid II is a validated, yet underutilized target for natural antibiotic compounds, with only vancomycin approved for clinical use. This is surprising, since compounds representing four different classes of natural compounds with no apparent structural similarity bind to Lipid II. Another class of natural compounds that bind to Lipid II has now been added with defensins. Even within defensins, there are seemingly no apparent structural or sequence motifs that determine Lipid II interactions. Defensins of both the alpha- and beta-families, classified by differences in cysteine connectivity and fold, reportedly bind Lipid II. Vertebrate as well as invertebrate defensins interact with Lipid II, as do defensins that are highly cationic (Human β-defensin 3, +11) [18], [19]. In this study, we have reported on the unique interaction of HNP-1 with Lipid II. Isoleucine at position 20 is found to bind Lipid II via multiple interactions. Recently, the structure of I20A has been solved and shows the usual HNP-1 fold (Zao *et. al.*, submitted for publication). The asymmetric unit of I20A-HNP1 crystal contains 2 monomers, but they were not arranged into dimers. Analysis of intermolecular contacts within the I20A-HNP1 crystal unambiguously ruled out the formation of any quaternary structure for the I20A-HNP1 mutant. This further supports the notion that for its Lipid II binding activity, HNP-1 functionally acts as a dimer, and, more importantly, that Isoleucine at position 20 is critical for Lipid II binding, since Lipid II binding and *S. aureus* killing, but not GP120 binding, anthrax lethal factor binding [50] or *E. coli* killing (unpublished), is abrogated by this mutation. Crystallographic analysis of the HNP-1-Lipid II complex unambiguously defined two HNP-1, but not three HNP-1 molecules which were arranged into wild-type HNP-1 dimer. Further, one, Lipid II molecule could be defined. Since interactions are described between Lipid II and residues of both monomers in the functional HNP-1 dimer, the stoichiometry between the two molecules of HNP-1 and one molecule of 3-Lipid II is arguably a 1∶1, and not a 2∶1 binding event. We did however observe that full inhibition of bacterial killing by Lipid II was not achieved at 1∶1 molar ratios. In these experiments, water-soluble 3-Lipid II was used for inhibition. It is conceivable that full inhibition of HNP-1 occurs in the context of the membrane environment, i.e. that the affinity of HNP-1 for membrane-bound Lipid II is higher than for its soluble form. In that case, one would need more than 1∶1 of soluble Lipid II for full functional inhibition. Besides that, alternative mechanisms cannot be excluded.

Elevated concentrations of HNP-1 were reported in plasma, blood and body fluids such as pleural fluid, bronchoalveolar lavage fluid, urine, and cerebrospinal fluid from patients with a variety of infections including bacterial and non-bacterial infections and pulmonary tuberculosis [57]. More recently, lower than normal levels of HNPs or inactivation of the peptides have been linked to an increased risk of caries in the oral cavity [58] as well as infections of the airways including cystic fibrosis [59], [60]. Although the concentration of HNP-1 inside granules of neutrophils has been estimated in the mg/ml range, its concentration in serum is only detectable in ng/ml ranges in these studies. Combined with inhibition of the antimicrobial activity of HNP-1 by the presence of salts or serum, it was never likely that HNP-1, and perhaps other defensins, could be practically developed as natural antibiotics.

Remarkably, no synthetic compounds that interfere with Lipid II have yet been developed. That is why we used the Lipid II binding footprint of HNP-1 to guide our search to identify low-molecular weight drug-like molecules that act as defensin mimics using CADD. Subsequent experimental characterization of these compounds showed several that show preferential activity against Gram-positive organisms while being non-toxic to host cells at comparable concentrations. One promising compound, BAS00127538, was further characterized. Defensin mimetic BAS00127538 targets the aminosugar moiety of the Lipid II molecule, thus making cross-resistance with vancomycin unlikely. In addition to modification of the pentapeptide, modifications in the aminosugar residues in Lipid II that make up the peptidoglycan subunit can cause resistance also for many Gram-positive pathogens [61]. Such modifications often involve chemical modifications such as acetylation or de-acetylation. Further, BAS00127538 primarily affects cell wall synthesis and shows *in vivo* protection of sepsis. A second compound identified in our search, 1499-1221, primarily disrupts the membrane as its mechanism-of-action. Results obtained with compounds like 1499-1221 and other defensin mimetic compounds will prove invaluable both for validation and optimization of leads such as BAS00127538. Studies like these on defensin mimetics and on plectasin may provide insight for future development, design and synthesis of efficient, defensin-derived compounds specifically targeting Lipid II as promising therapeutic leads. To our knowledge, BAS00127538 is the first low-molecular weight compound that targets Lipid II that has been identified.

## Supporting Information

Figure S1 **Stereoview of a fit of HNP-1 dimer to the electron density map from experimental phases.** The protein residues of HNP-1 build into the model are shown in ball-and-stick representation. The *F*_o_ − *F*_c_ electron density omit maps are contoured at the 3 ó level (green), whereas the 2*F*_o_ − *F*_c_ electron density maps are contoured at the 1ó level (blue). The picture was generated using Coot (Emsley, P., and Cowtan, K. (2004) *Acta Crystallogr D Biol Crystallogr* **60**, 2126-2132).
(PDF)

Figure S2 **Structural alignment of dimers as observed in crystals grown from HNP1-Lipid II mixture (yellow) and wild-type HNP-1 (green, (PDB:1GNY)).** Dimers were aligned based on monomer A and only residues identified with HADDOCK docking model as Lipid II contacts residues are shown as ball-sticks and colored in red. Pairwise superimposition analysis of HNP-1 alone or HNP-1 in complex with Lipid II revealed very close similarity as shown by average RMDS value of 0.8 Å for 60 aligned Cá atoms. Although the overall structure of dimers is the same, their pairwise superimposition indicates an apparent shift of the monomer B backbone forming *β*1/*β*2 and *β*2/*β*3 connecting loops and the *β*3 strand.
(PDF)

Figure S3 **Detailed superimposition of residues identified by HADDOCK to be involved in Lipid II interactions.** In the dimer of crystals grown from HNP-1-lipid II mixture (yellow) the backbone atom of L_b_25 and R_b_15 shift around 2.0 Å and 1.3 Å, respectively, toward the lipid II binding site as compared to wild-type HNP-1 (green, (PDB:1GNY).
(PDF)

Figure S4 **Mechanism of action studies of 1499-1221.** Exponentially growing *S. aureus 29213* cells were exposed to compound and comparators in triplicate using 2.5% DMSO as “no drug” control. Cells were added to Mueller-Hinton Broth or M9 medium for protein synthesis and further incubated in the presence of [^14^C]N-acetyl glucosamine (cell wall), [^3^H]glycerol (lipid), [^3^H]Thymidine (DNA), or [^3^H]Leucine (protein). Following incubation, reactions were stopped by adding TCA (DNA, protein), 8% SDS (cell wall) or chloroform/methanol (lipid) and analyzed by scintillation counting.
(PDF)

Table S1 **Data collection and refinement statistics.**
(PDF)

Table S2 **Cluster statistics of the HADDOCK docking run calculated on the top 4 member of each cluster.**
(PDF)

Table S3 **Summary of defensin mimetic compounds.**
(PDF)
