# Correction: Turning Defense into Offense: Defensin Mimetics as Novel Antibiotics Targeting Lipid II

**DOI:** 10.1371/journal.ppat.1004611

**Published:** 2014-12-18

**Authors:** The PLOS Pathogens Staff

Table S3 in the published article contains an error. One compound, 4890-0291, has been incorrectly classified as not binding to Lipid II. The authors have confirmed binding of this compound to Lipid II by NMR. The corrected version of Table S3 can be viewed here.

## Supporting Information

Table S3 **Summary of defensin mimetic compounds.**
(PDF)
