# Supplementary figures and images for: Turning Defense into Offense: Defensin Mimetics as Novel Antibiotics Targeting Lipid II
Source: PLoS Pathog. 2013 Nov 7;9(11):e1003732. doi: 10.1371/journal.ppat.1003732 (PMC3820767; doi:10.1371/journal.ppat.1003732)

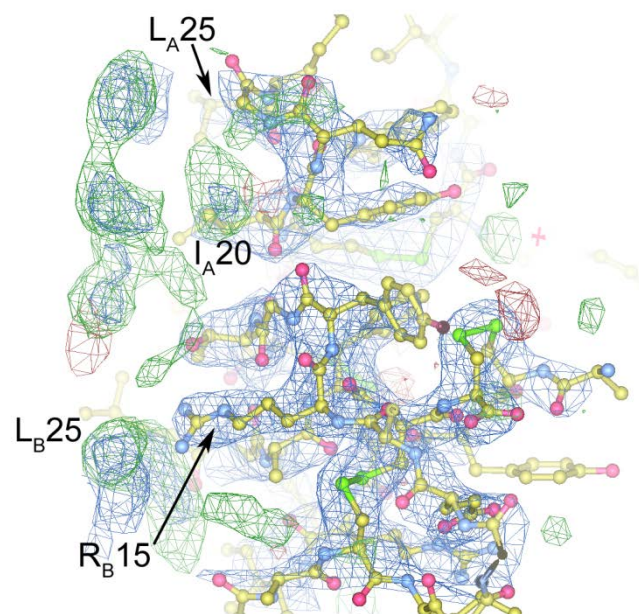

Supplement: Figure S1 — Stereoview of a fit of HNP-1 dimer to the electron density map from experimental phases. The protein residues of HNP-1 build into the model are shown in ball-and-stick representation. The F o − F c electron density omit maps are contoured at the 3 ó level (green), whereas the 2F o − F c electron density maps are contoured at the 1ó level (blue). The picture was generated using Coot (Emsley, P., and Cowtan, K. (2004) Acta Crystallogr D Biol Crystallogr 60, 2126-2132). (PDF) [file ppat.1003732.s001.pdf]

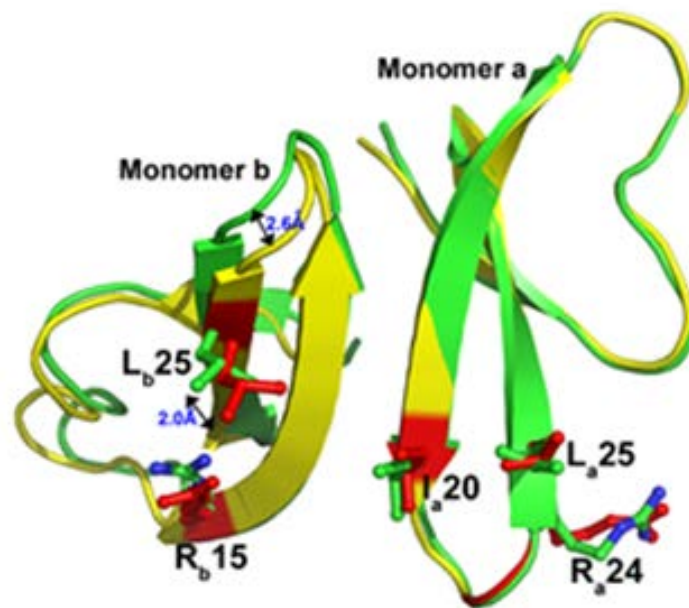

Supplement: Figure S2 — Structural alignment of dimers as observed in crystals grown from HNP1-Lipid II mixture (yellow) and wild-type HNP-1 (green, (PDB:1GNY)). Dimers were aligned based on monomer A and only residues identified with HADDOCK docking model as Lipid II contacts residues are shown as ball-sticks and colored in red. Pairwise superimposition analysis of HNP-1 alone or HNP-1 in complex with Lipid II revealed very close similarity as shown by average RMDS value of 0.8 Å for 60 aligned Cá atoms. Although the overall structure of dimers is the same, their pairwise superimposition indicates an apparent shift of the monomer B backbone forming β1/β2 and β2/β3 connecting loops and the β3 strand. (PDF) [file ppat.1003732.s002.pdf]

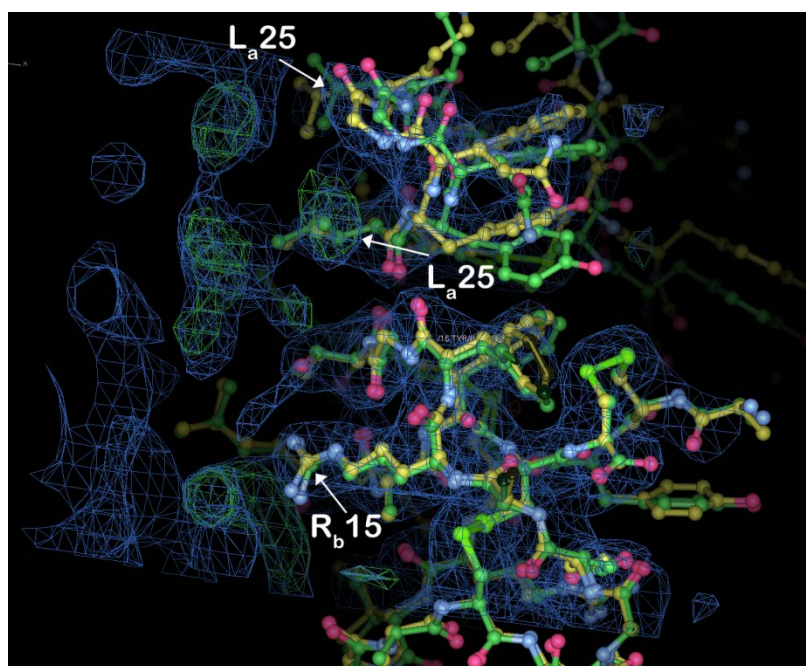

Supplement: Figure S3 — Detailed superimposition of residues identified by HADDOCK to be involved in Lipid II interactions. In the dimer of crystals grown from HNP-1-lipid II mixture (yellow) the backbone atom of Lb25 and Rb15 shift around 2.0 Å and 1.3 Å, respectively, toward the lipid II binding site as compared to wild-type HNP-1 (green, (PDB:1GNY). (PDF) [file ppat.1003732.s003.pdf]

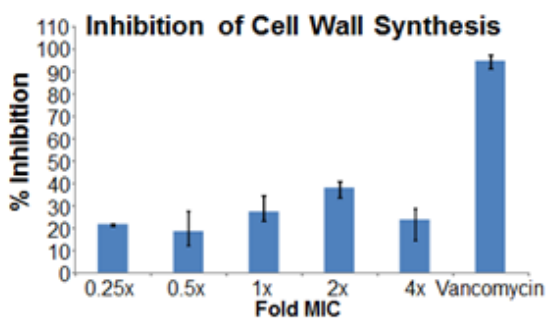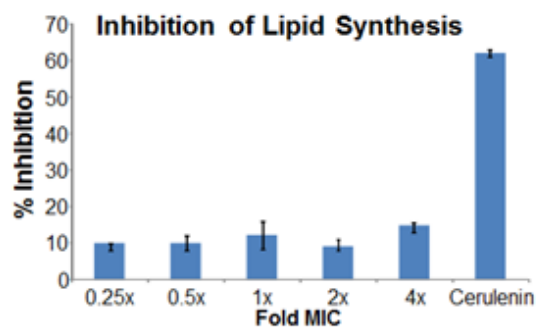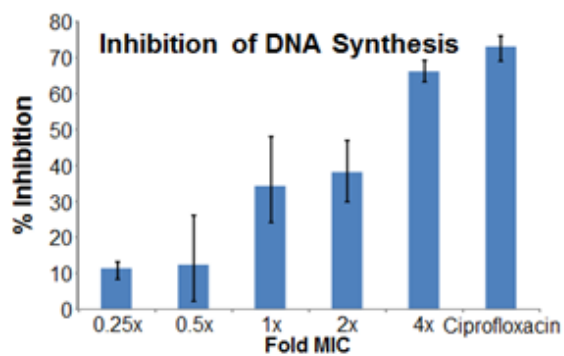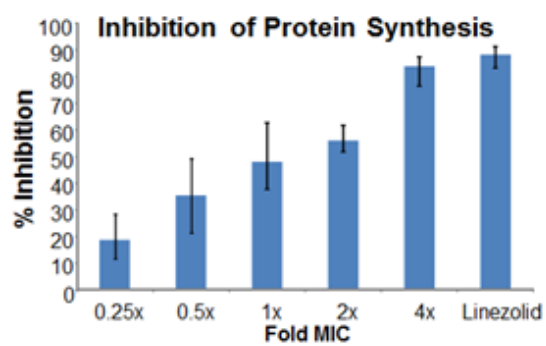

Supplement: Figure S4 — Mechanism of action studies of 1499-1221. Exponentially growing S. aureus 29213 cells were exposed to compound and comparators in triplicate using 2.5% DMSO as “no drug” control. Cells were added to Mueller-Hinton Broth or M9 medium for protein synthesis and further incubated in the presence of [14C]N-acetyl glucosamine (cell wall), [3H]glycerol (lipid), [3H]Thymidine (DNA), or [3H]Leucine (protein). Following incubation, reactions were stopped by adding TCA (DNA, protein), 8% SDS (cell wall) or chloroform/methanol (lipid) and analyzed by scintillation counting. (PDF) [file ppat.1003732.s004.pdf]
