# Supplementary material for: Turning Defense into Offense: Defensin Mimetics as Novel Antibiotics Targeting Lipid II
Source: PLoS Pathog. 2013 Nov 7;9(11):e1003732. doi: 10.1371/journal.ppat.1003732 (PMC3820767; doi:10.1371/journal.ppat.1003732)
Supplement: Table S1 — Data collection and refinement statistics. (PDF) [file ppat.1003732.s005.pdf]

Data Collection, Structure Solution, and Refinement

Prior to data collection, the crystal was rapidly soaked in well-solution containing 5% v/v glycerol and flash-cooled to 100K in a nitrogen stream. X-ray diffraction data were collected using a rotating anode X-ray generator Rigaku-MSC Micromax 7 and a Raxis-4++ image plate detector (at the X-ray Crystallography Core Facility, University of Maryland, Baltimore) and were integrated and scaled with the HKL2000 package (1). Structure was solved by the molecular replacement method with program Phaser from the CCP4 suite (2), using model based on the monomer of HNP-1 (PDB:1GNY, (3)) a probe (4) and refined with program Refmac and coupled with manual refitting and rebuilding with COOT (5). Data collection and refinement statistics are summarized in Table1. Molecular graphics were generated using Pymol (http://pymol.org).

Supplementary Table 1. Data collection and refinement statistics

Space group	I432
Cell parameters, Å	a=107.0, b=107.0, c=107.0
Molecules/a.u.	2
Resolution, Å	50-2.55 (2.59-2.55)
Number of reflections	
Total	580,408
Unique	6,472
R_{merge}[b], %	11.1 (7.4)
Completeness, %	99.8 (99.8)
Redundancy	8.8 (8.9.)
I/σ, (I)	19.3 (2.3)
Resolution, Å	20-2.55
R[c], %	20.8
R_{free}[d], %	23.6
No. of protein atoms	476
No. of solvent molecules	15
No. of heteroatoms	17
Root mean square deviation	
Bond lengths, Å	0.015
Bond angles, °	1.54

[a]All data (outer shell).

[b]$R_{merge} = \sum | I - \langle I \rangle | / \sum I$, where *I* is the observed intensity and <*I*> is the average intensity obtained from multiple observations of symmetry-related reflections after rejections

[c]$R = \sum \| F_o | - | F_c \| / \sum | F_o |$, where F_o and F_c are the observed and calculated structure factors, respectively

[d]R_{free} = defined by by Brünger (6)

1. Otwinowski, Z., Minor, W., and Charles W. Carter, Jr. (1997) Processing of X-ray diffraction data collected in oscillation mode. in *Methods in Enzymology*, Academic Press. pp 307-326
2. (1994) *Acta Crystallogr D Biol Crystallogr* **50**, 760-763
3. Wei, G., de Leeuw, E., Pazgier, M., Yuan, W., Zou, G., Wang, J., Ericksen, B., Lu, W. Y., Lehrer, R. I., and Lu, W. (2009) *J Biol Chem* **284**, 29180-29192
4. Szyk, A., Wu, Z., Tucker, K., Yang, D., Lu, W., and Lubkowski, J. (2006) *Protein Sci* **15**, 2749-2760
5. Emsley, P., and Cowtan, K. (2004) *Acta Crystallogr D Biol Crystallogr* **60**, 2126-2132
6. Brunger, A. T. (1992) *Nature* **355**, 472-475
