# Supplementary material for: Turning Defense into Offense: Defensin Mimetics as Novel Antibiotics Targeting Lipid II
Source: PLoS Pathog. 2013 Nov 7;9(11):e1003732. doi: 10.1371/journal.ppat.1003732 (PMC3820767; doi:10.1371/journal.ppat.1003732)
Supplement: Table S2 — Cluster statistics of the HADDOCK docking run calculated on the top 4 member of each cluster. (PDF) [file ppat.1003732.s006.pdf]

Supplementary Table 2. Cluster statistics of the HADDOCK docking run calculated on the top 4 members of each cluster.

	**Cluster 1**	**Cluster 2**
Cluster rank	1	1
Cluster population	296	103
RMSD from overall lowest-energy structure (Å)	1.25 ± 0.72	4.07 ± 0.07
HADDOCK score[a] (a.u.)	-35.7 ± 0.1	-26.4 ± 2.0
Intermolecular van der Waals energy[b] (E_{vdw})(kcal mol^{-1})	-18.9 ± 5.0	-14.7 ± 2.4
Intermolecular electrostatic energy[b] (E_{elec}) (kcal mol^{-1})	-69.9 ± 15.4	-61.5 ± 7.6
Desolvation energy[c] (E_{desol}) (kcal mol^{-1})	-3.4 ± 2.7	-0.8 ± 1.5
Restraints violation energy (E_{AIR}) (kcal mol^{-1})	0.6 ± 0.2	1.4 ± 0.8
Buried surface area ($Å^2$)	646 ± 71	649 ± 32

a. The HADDOCK score is defined as: 1.0 E_{vdw} + 0.2 E_{elec} + 1.0 E_{desol} + 0.1 E_{AIR}

b. Non-bonded interactions were calculated with the OPLS force field (Jorgensen W, Tirado-Rives J. The OPLS potential functions for proteins. Energy minimizations for crystals of cyclic peptides and crambin. J Am Chem Soc 1988;110(6):1657–1666) using a 8.5Å cut-off.

c. Calculated using the empirical desolvation energy parameters from Fernandez-Recio et al (Fernández-Recio J, Totrov M, Abagyan R. Identification of protein-protein interaction sites from docking energy landscapes. J Mol Biol 2004;335(3):843–865).
