# Supplementary material for: Turning Defense into Offense: Defensin Mimetics as Novel Antibiotics Targeting Lipid II
Source: PLoS Pathog. 2013 Nov 7;9(11):e1003732. doi: 10.1371/journal.ppat.1003732 (PMC3820767; doi:10.1371/journal.ppat.1003732)
Supplement: Table S3 — Summary of defensin mimetic compounds. (PDF) [file ppat.1003732.s007.pdf]

ID	structural formula	chemical formula	colored?	MW (g/ mol)	IUPAC name	MFR	Binding on Lipid II?	active against S.aureus	active against E.coli	cytotoxic for Caco II ($C_{50\% \text{ survival}}$)	cytotoxic for Jurkat ($C_{50\% \text{ survival}}$)	compound category	Nr.	
5457685	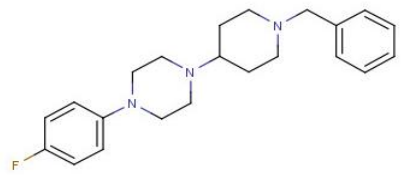	$C_{22}H_{28}FN_3$	no	353.5	1-(1-benzyl-4-piperidinyl)-4-(4-fluorophenyl) piperazine	Chem Bridge	No	No	No	No	?	A	1	
5452876	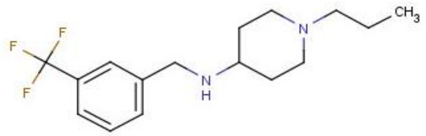	$C_{16}H_{23}F_3N_2$	no	300.4	1-propyl-N-[3-(trifluoromethyl)benzyl]-4-piperidinamine	Chem Bridge	No	No	No	No	?	A	2	
5270046	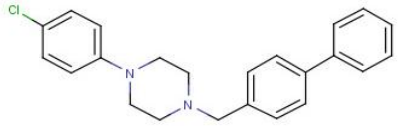	$C_{23}H_{23}ClN_2$	no	362.9	1-(4-biphenylylmethyl)-4-(4-chlorophenyl) piperazine	Chem Bridge	No	No	No	No	?	A	3	
6711103	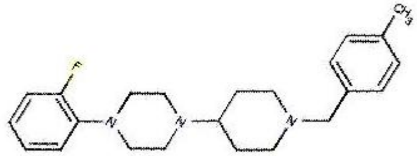	$C_{23}H_{30}FN_3$	no	367.5	1-(2-fluorophenyl)-4-[1-(4-methylbenzyl)-4-piperidinyl]piperazine	Chem Bridge	No	No	No	No	?	A	4	plate A
5418391	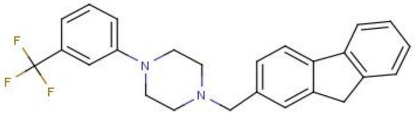	$C_{25}H_{23}F_3N_2$	no	408.5	1-(9H-fluoren-2-ylmethyl)-4-[3-(trifluoromethyl)phenyl] piperazine	Chem Bridge	No	No	No	Yes (159)	?	A	5	

5267798	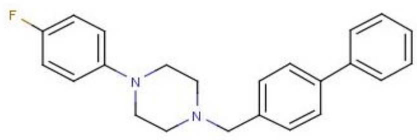	$C_{23} H_{23} F N_2$	no	346.4	1-(4-biphenylylmethyl)-4-(4-fluorophenyl) piperazine	Chem Bridge	No	No	No	No	?	A	6	
6785396	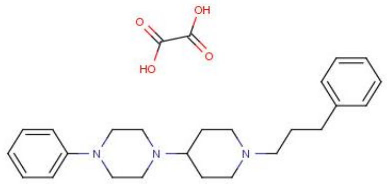	$C_{24} H_{33} N_3$. $C_2 H_2 O_4$	no	453.6	1-phenyl-4-[1-(3-phenylpropyl)-4-piperidinyl] piperazine oxalate	Chem Bridge	No	No	No	Yes (168)	?	A	7	plate B
5422765	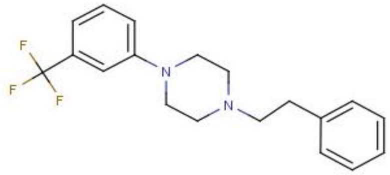	$C_{19} H_{21} F_3 N_2$	no	334.4	1-(2-phenylethyl)-4-[3-(trifluoromethyl) phenyl] piperazine	Chem Bridge	Yes	No	No	Yes (130)	?	A*	8	
5230300	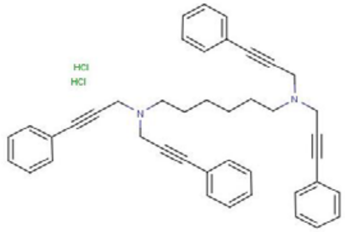	$C_{42} H_{40} N_2$. 2 Cl H	no	645.7	N,N,N',N'-tetrakis (3-phenyl-2-propyn-1-yl)-1,6-hexanediamine dihydrochloride	Chem Bridge	No	No	No	No	?	A	9	
5230313	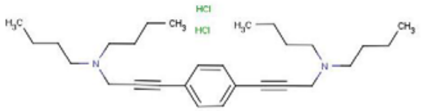	$C_{28} H_{44} N_2$. 2 Cl H	no	481.6	3,3'-(1,4-phenylene) bis (N,N-dibutyl-2-propyn-1-amine) dihydrochloride	Chem Bridge	Yes	No	No	Yes (366)	?	A*	10	

7771-0701	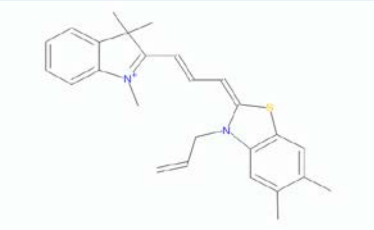	$C_{26} H_{29} N_2 S$	yes	401.6	n/a	Chem Div	Yes	Yes (15.625)	Yes (31.25)	Yes (16,7)	?	B/C*	11	
8006-3639	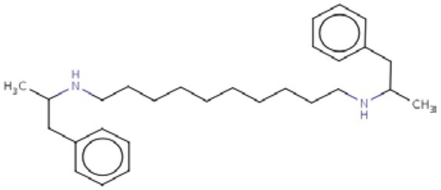	$C_{28} H_{44} N_2$	no	408.7	n/a	Chem Div	No	Yes (125)	Yes (125)	Yes (3,6)	?	B	12	
1499-1221	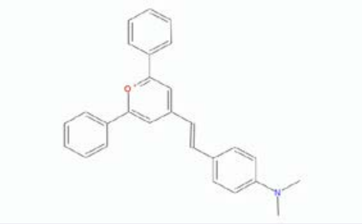	$C_{27} H_{24} N O$	yes	378.5	n/a	Chem Div	No	Yes (1.95)	Yes (125)	Yes (8,9)	?	B/C	13	
5429346	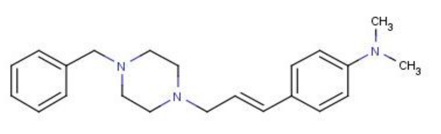	$C_{22} H_{29} N_3$	(yes)	335.5	4-[3-(4-benzyl-1-piperazinyl)-1-propen-1-yl]-N,N-dimethylaniline	Chem Bridge	Yes	Yes (500)	No	Yes (131,9)	?	C*	14	
0251-0215	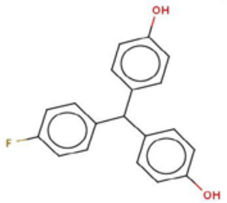	$C_{19} H_{15} F O_2$	no	294.3	n/a	Chem Div	No	Yes (125)	No	Yes (17,1)	?	B/C	15	
5427129	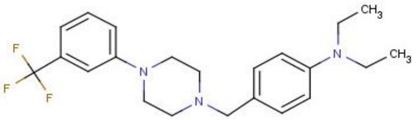	$C_{22} H_{28} F_3 N_3$	no	391.5	N,N-diethyl-4-({4-[3-(trifluoromethyl)phenyl]-1-piperazinyl}methyl) aniline	Chem Bridge	Yes	(No)	No	Yes (192,2)	?	A*	16	plate C

4636-0141	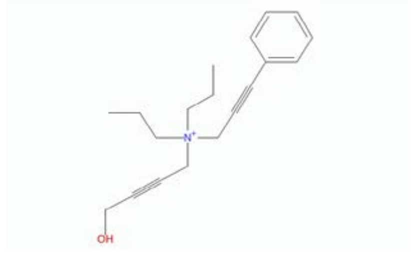	$C_{19}H_{26}NO$	no	284.4	(4-hydroxybut-2-yn-1-yl)(3-phenylprop-2-yn-1-yl)dipropylazanium	Chem Div	No	No	No	No	?	A	17	
5100015	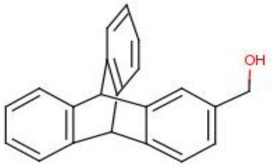	$C_{21}H_{16}O$	no	284.4	pentacyclo[6.6.6.0~2,7~.0~9,14~.0~15,20~]icosa-2,4,6,9,11,13,15,17,19-nonaen-4-ylmethanol	Chem Bridge	No	Yes (500)	No	Yes (68)	?	B/C	18	
0438-0367	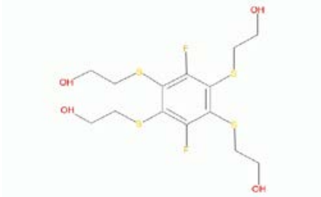	$C_{14}H_{20}F_2O_4S_4$	no	418.6	2-({2,5-difluoro-3,4,6-tris[(2-hydroxyethyl)sulfanyl]phenyl}sulfanyl)ethan-1-ol	Chem Div	No	No	No	No	?	A	19	
N050-0022	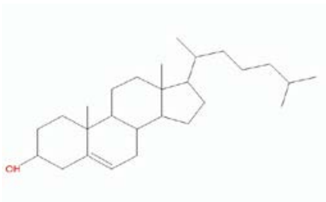	$C_{27}H_{46}O$	no	386.7	2,15-dimethyl-14-(6-methylheptan-2-yl)tetracyclo[8.7.0.0^{2,7}.0^{11,15}]heptadec-7-en-5-ol	Chem Div	No	No	No	Yes (122,9)	?	A	20	
5607-0206	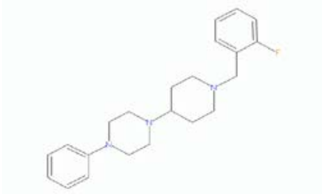	$C_{22}H_{28}FN_3$	no	353.5	1-{1-[(2-fluorophenyl)methyl]piperidin-4-yl}-4-phenylpiperazine	Chem Div	No	No	No	No	?	A	21	ate D

5400-0379	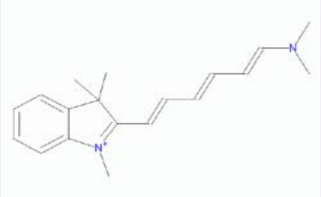	$C_{19} H_{25} N_2$	yes	281.4	n/a	Chem Div	No	Yes (125)	No	Yes (55,8)	?	B/C	22	pl
2729-1114	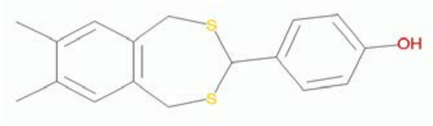	$C_{17} H_{18} O S_2$	no	302.5	4-(7,8-dimethyl-3,5-dihydro-1H-2,4-benzodithiepin-3-yl) phenol	Chem Div	No	No	No	Yes (119,1)	?	A	23	
000A-0859	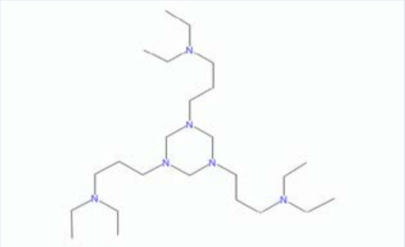	$C_{24} H_{54} N_6$	no	426.7	(3-{3,5-bis [3-(diethylamino)propyl]-1,3,5-triazinan-1-yl}propyl) diethylamine	Chem Div	Yes	Yes (500)	Yes (500)	Yes (418,6)	?	B*	24	
5210734	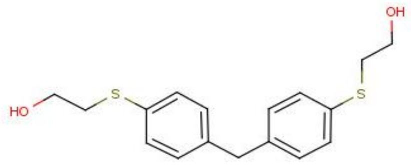	$C_{17} H_{20} O_2 S_2$	no	320.5	2,2'-[methylenebis (4,1-phenylenethio)] diethanol	Chem Bridge	No	No	No	No	?	A	25	plate E (+ DMSO)
5230234	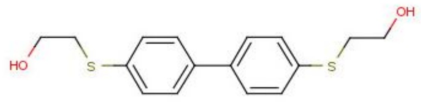	$C_{16} H_{18} O_2 S_2$	no	306.4	2,2'-[4,4'-biphenyldiylbis(thio)] diethanol	Chem Bridge	No	No	No	No	?	A	26	
5460400	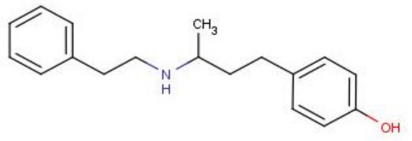	$C_{18} H_{23} N O$	no	269.4	4-{3-[(2-phenylethyl) amino]butyl} phenol	Chem Bridge	No	No	No	No	?	A	27	
7191067	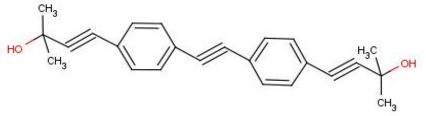	$C_{24} H_{22} O_2$	no	342.4	4,4'-(1,2-ethynediyldi-4,1-phenylene) bis(2-methyl-3-butyn-2-ol)	Chem Bridge	No	No	No	No	?	A	28	

7411164	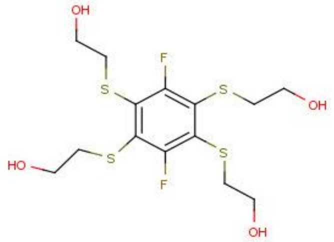	$C_{14}H_{20}F_2O_4S_4$	no	418.6	2,2',2'',2'''-[(3,6-difluorobenzene-1,2,4,5-tetrayl)tetrakis (thio)]tetraethanol	Chem Bridge	No	No	No	No	?	A	29	
4431/1	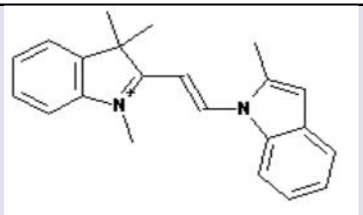	$C_{22}H_{23}N_2$	No	315.4	1,3,3-trimethyl-2-(2-(2-methyl-1H-indol-1-yl) vinyl)-3H-1λ^5-indole	NCI	No	Yes (250)	No	No	?	C	31	
55870/1	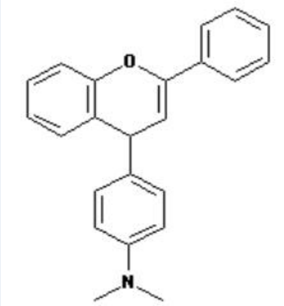	$C_{23}H_{21}NO$	No	327.4	N,N-dimethyl-4-(2-phenyl-4H-chromen-4-yl) aniline	NCI	Yes	Yes (250)	No	Yes (25,9)	?	C*	32	
35487/2	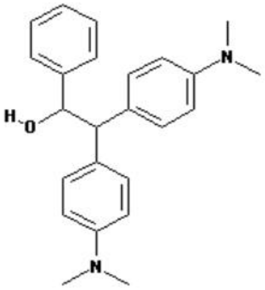	$C_{24}H_{28}N_2O$	No	360.5	2,2-bis (4-(dimethylamino)phenyl)-1-phenylethanol	NCI	No	No	No	No	?	A	33	plate 1

96932/4	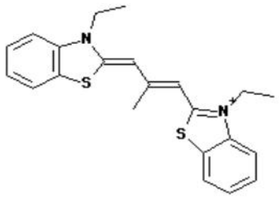	$C_{22}H_{23}N_2S_2$	Yes	379.6	3-ethyl-2-(3-(3-ethyl-1,3-benzothiazol-2(3H)-ylidene)-2-methyl-1-propenyl)-1,3λ^5-benzothiazole	NCI	No	Yes (15,6)	Yes (15.625)	Yes (19)	?	B	34
625586/1	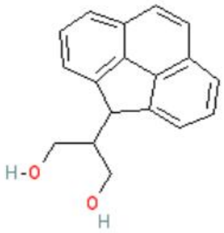	$C_{18}H_{16}O_2$	No	264.3	2-(4H-cyclopenta[def] phenanthren-4-yl)-1,3-propanediol	NCI	No	No	No	Yes (83,8)	?	A	35
610995/1	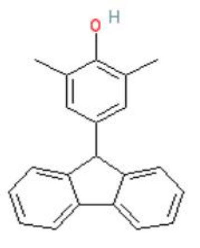	$C_{21}H_{18}O$	No	286.4	4-(9H-fluoren-9-yl)-2,6-dimethylphenol	NCI	Yes	Yes (125)	No	Yes (9,2)	?	B/C*	36
290437/2	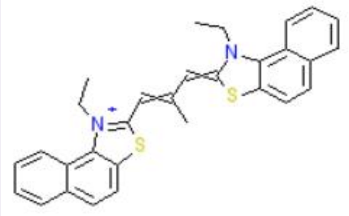	$C_{30}H_{27}N_2S_2$	Yes	479.7	1-ethyl-2-(3-(1-ethylnaphtho[1,2-d] [1,3]thiazol-2(1H)-ylidene)-2-methyl-1-propenyl)-1λ^5-naphtho[1,2-d][1,3] thiazole	NCI	No	Yes (1,95)	Yes (31.35)	Yes (2,2)	?	B/C	37
4090-1978	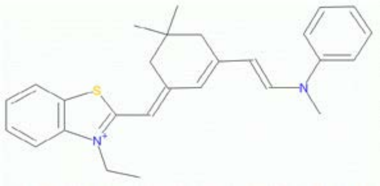	$C_{27}H_{31}N_2S$	Yes	415.6	2-{[(1E)-5,5-dimethyl-3-[(E)-2-[methyl(phenyl) amino]ethenyl] cyclohex-2-en-1-ylidene] methyl}-3-ethyl-1,3-benzothiazol-3-ium	Chem Div	No	Yes (15,625)	Yes (62,5)	Yes (16,7)	?	B/C	38

2332-1014	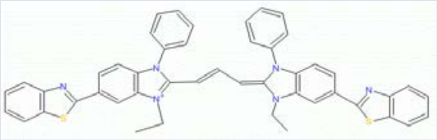	$C_{47}H_{37}N_6S_2$	Yes	750.0	n/a	Chem Div	No	Yes (62,5)	No	Yes (22,6)	?	B/C	39
4890-0291	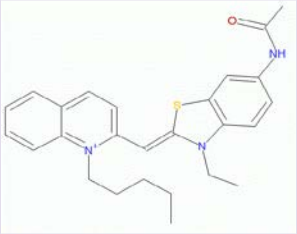	$C_{26}H_{30}N_3OS$	Yes	432.6	n/a	Chem Div	No	Yes (7,81)	Yes (62,5)	Yes (10,7)	?	B/C	40
4121-0081	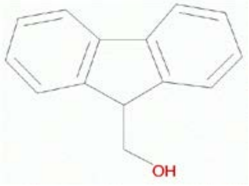	$C_{14}H_{12}O$	No	196.2	9H-fluoren-9-ylmethanol	Chem Div	No	No	No	Yes (201,8)	?	A	41
C611-0808	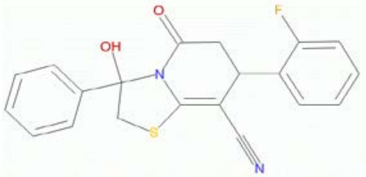	$C_{20}H_{15}FN_2O_2S$	No	366.4	n/a	Chem Div	No	No	No	No	?	A	42
3377-0105	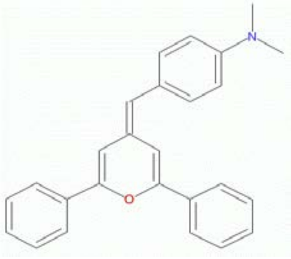	$C_{26}H_{23}NO$	No	365.5	4-[(2,6-diphenyl-4H-pyran-4-ylidene)methyl]-N,N-dimethylaniline	Chem Div	No	Yes (500)	No	Yes (100,4)	?	A/B	43

plate 2

2331-0475	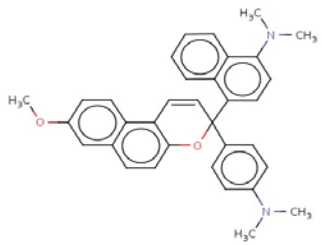	$C_{34}H_{32}N_2O_2$	No	500.6	n/a	Chem Div	No	No	No	No	?	A	44	
7771-0699	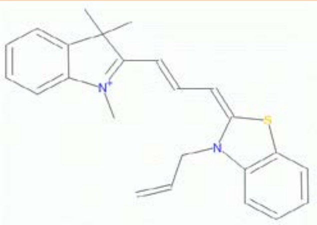	$C_{24}H_{25}N_2S$	Yes	373.5	n/a	Chem Div	No	Yes (7,8)	Yes (125)	Yes (7,9)	?	B	45	plate 3
7771-0716	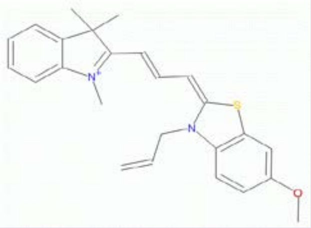	$C_{25}H_{27}N_2OS$	Yes	403.6	n/a	Chem Div	No	Yes (15,625)	Yes (125)	Yes (5,3)	?	B	46	
7771-0700	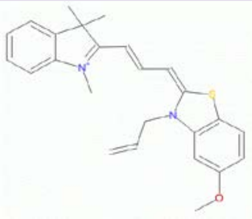	$C_{25}H_{27}N_2OS$	Yes	403.6	n/a	Chem Div	No	Yes (7,8)	Yes (62,5)	Yes (3,7)	?	B	47	
7165-0606	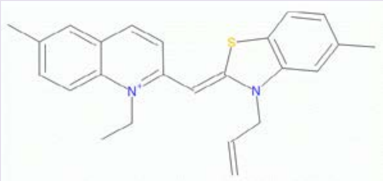	$C_{24}H_{25}N_2S$	Yes	373.5	n/a	Chem Div	No	Yes (7,8)	Yes (31,25)	Yes (11,9)	?	B	48	

7165-0758	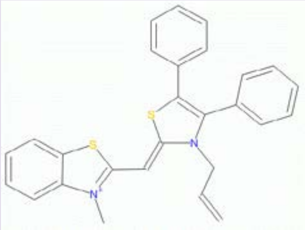	$C_{27} H_{23} N_2 S_2$	No	439.6	n/a	Chem Div	No	Yes (7,8)	Yes (125)	Yes (6,7)	?	B	49	
1492-0330	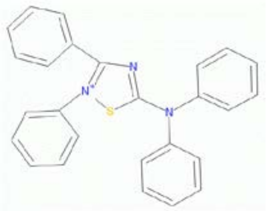	$C_{26} H_{20} N_3 S$	No	406.5	5-(diphenylamino)-2,3-diphenyl-1,2$l^{5},4-thiadiazol-2-ylium	Chem Div	Yes	No	No	Yes (101,2)	?	A*	50	
2101-0102	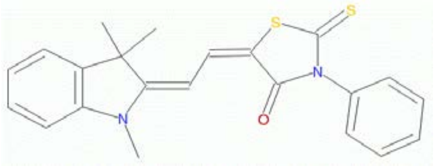	$C_{22} H_{20} N_2 O S_2$	Yes	392.5	(5E)-3-phenyl-2-sulfanylidene-5-{2-[(2E)-1,3,3-trimethyl-2,3-dihydro-1H-indol-2-ylidene]ethylidene}-1,3-thiazolidin-4-one	Chem Div	No	No	No	No	?	A	51	
1611-0203	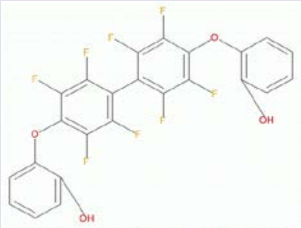	$C_{24} H_{10} F_8 O_4$	No	514.3	2-{2,3,5,6-tetrafluoro-4-[2,3,5,6-tetrafluoro-4-(2-hydroxyphenoxy)phenyl]phenoxy}phenol	Chem Div	No	Yes (3,9)	No	Yes (4,2)	?		52	plate 4
4896-3423	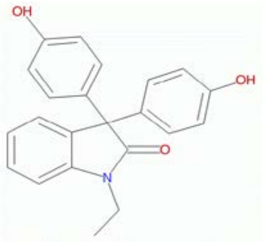	$C_{22} H_{19} N O_3$	No	345.4	1-ethyl-3,3-bis(4-hydroxyphenyl)-2,3-dihydro-1H-indol-2-one	Chem Div	No	No	No	Yes (72,1)	?	A	53	

5660386	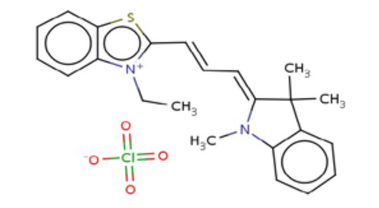	C_{23} H_{25} Cl N_2 O_4 S	Yes	461.0	3-ethyl-2-[(1E)-3-[(2Z)-1,3,3-trimethyl-2,3-dihydro-1H-indol-2-ylidene]prop-1-en-1-yl]-1,3-benzothiazol-3-ium perchlorate	Chem Bridge	No	Yes (7,8)	Yes (125)	Yes (5,6)	?		54	
STK 874226	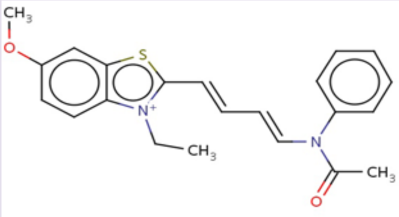	$C_{22}H_{23}N_2O_2S$	Yes	379.5	2-{(1E,3E)-4-[acetyl(phenyl)amino] buta-1,3-dien-1-yl}-3-ethyl-6-methoxy-1,3-benzothiazol-3-ium	VitasM Lab	No	Yes (125)	Yes (250)	No	?		55	
5119716	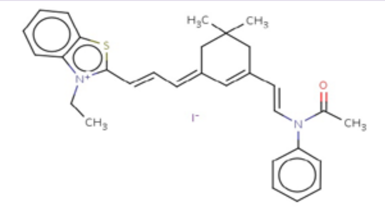	C_{30} H_{33} I N_2 O S	Yes	596.6	2-[(1E)-3-[(1E)-5,5-dimethyl-3-[(E)-2-(N-phenylacetamido) ethenyl]cyclohex-2-en-1-ylidene]prop-1-en-1-yl]-3-ethyl-1,3-benzothiazol-3-ium iodide	Chem Bridge	No	Yes (125)	No	Yes (52,7)	?		56	
5100004	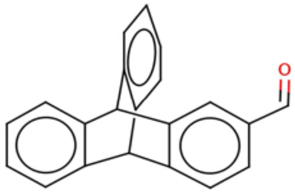	C_{21} H_{14} O	No	282.3	pentacyclo[6.6.6.0^{2,7}.0^{9,14}.0^{15,20}]icosa-2,4,6,9,11,13,15,17,19-nonaene-4-carbaldehyde	Chem Bridge	No	No	No	No	?	A	57	
5190620	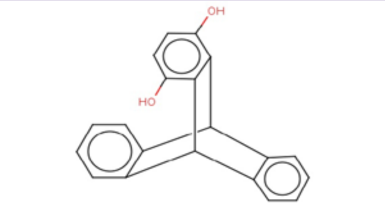	C_{20} H_{14} O_2	(Yes)	286.3	pentacyclo[6.6.6.0^{2,7}.0^{9,14}.0^{15,20}]icosa-2(7),3,5,9,11,13,15,17,19-nonaene-3,6-diol	Chem Bridge	No	Yes (62,5)	No	Yes (3,7)	?		58	plate 5

5142587	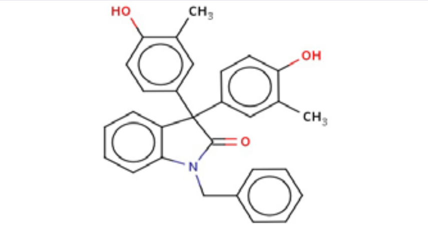	$C_{29} H_{25} N O_3$	No	435.5	1-benzyl-3,3-bis (4-hydroxy-3-methylphenyl)-2,3-dihydro-1H-indol-2-one	Chem Bridge	No	Yes (15,625)	No	Yes (4,4)	?		59
7832238	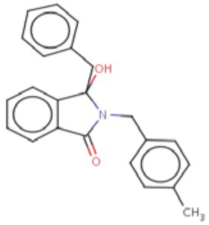	$C_{23} H_{21} N O_2$	No	343.4	3-benzyl-3-hydroxy-2-[(4-methylphenyl)methyl]-2,3-dihydro-1H-isoindol-1-one	Chem Bridge	No	No	No	No	?	A	60
BAS 00138032	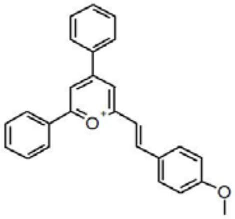	$C_{26} H_{21} O_2$	Yes	365.4	2-[2-(4-Methoxy-phenyl)-vinyl]-4,6-diphenyl-pyranylium	Asinex	No	No	No	No	?	A	61
BAS 00691248	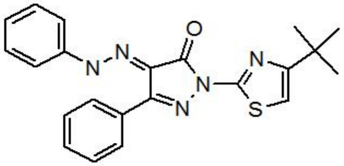	$C_{22} H_{21} N_5 O S$	Yes	403.5	2-(4-tert-Butyl-thiazol-2-yl)-5-phenyl-4-(phenyl-hydrazono)-2,4-dihydro-pyrazol-3-one	Asinex	No	No	No	No	?	A	62
SYN 22879441	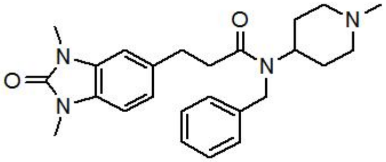	$C_{25} H_{32} N_4 O_2$	No	420.6	N-Benzyl-3-(1,3-dimethyl-2-oxo-2,3-dihydro-1H-benzoimidazol-5-yl)-N-(1-methyl-piperidin-4-yl)-propionamide	Asinex	No	No	No	No	?	A	63

ST 4098270	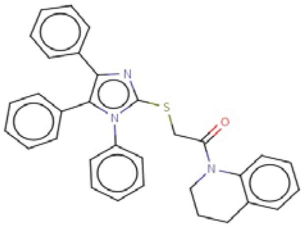	$C_{32} H_{27} N_3 O S$	No	501.7	1-(1,2,3,4-tetrahydroquinolin-1-yl)-2-[(1,4,5-triphenyl-1H-imidazol-2-yl)sulfanyl]ethan-1-one	Tim Tec	No	No	No	No	?	A	64	plate
STK 792971	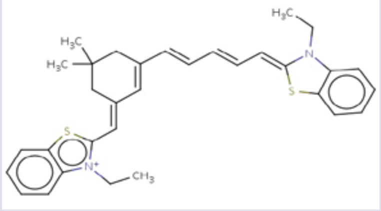	$C_{32} H_{35} N_2 S_2$	Yes	511.8	3-ethyl-2-[(E)-{3-[(1E,3E,5Z)-5-(3-ethyl-1,3-benzothiazol-2(3H)-ylidene)penta-1,3-dien-1-yl]-5,5-dimethylcyclohex-2-en-1-ylidene}methyl]-1,3-benzothiazol-3-ium	VitasM Lab	No	Yes (62,5)	No	No	?	C	65	
STK 537076	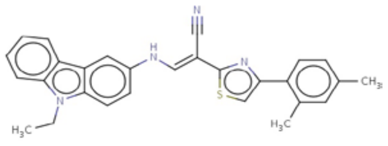	$C_{28} H_{24} N_4 S$	No	448.6	(2E)-2-[4-(2,4-dimethylphenyl)-1,3-thiazol-2-yl]-3-[(9-ethyl-9H-carbazol-3-yl)amino]prop-2-enenitrile	VitasM Lab	No	No	No	Yes (240)	?	A	66	
STL050072	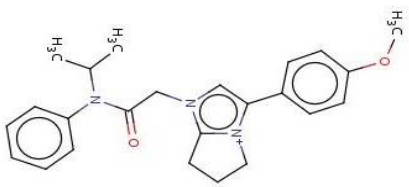	$C_{24} H_{28} N_3 O_2$	No	390.5	3-(4-methoxyphenyl)-1-{2-oxo-2-[phenyl(propan-2-yl)amino]ethyl}-1,5,6,7-tetrahydropyrrolo[1,2-a]imidazol-4-ium	VitasM Lab	No	No	No	Yes (340)	?	A	67	
STK 368153	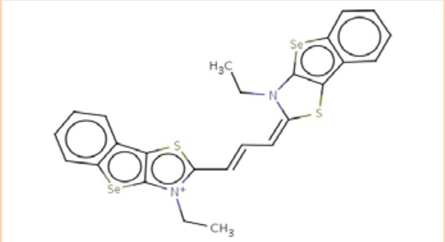	$C_{25} H_{21} N_2 S_2 Se_2$	Yes	571.5	3-ethyl-2-[(1E,3E)-3-(3-ethyl[1]benzoselenopheno[2,3-d][1,3]thiazol-2(3H)-ylidene)prop-1-en-1-yl][1]benzoselenopheno[2,3-d][1,3]thiazol-3-ium	VitasM Lab	Yes	Yes (7,8)	Yes (7,8)	Yes (9,6)	?	B*	68	

179415/1	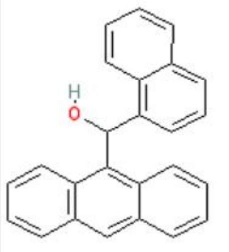	$C_{25}H_{18}O$	No	334.4	9-anthryl(1-naphthyl)methanol	NCI	Yes	Yes (3,9)	No	No	?	C*	69	plate 7
363003/1	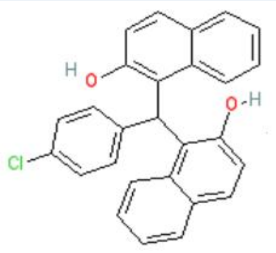	$C_{27}H_{19}ClO_2$	No	410.9	n/a	NCI	Yes	Yes (3,9)	No	Yes (3,5)	?	B/C*	70	
55266/1	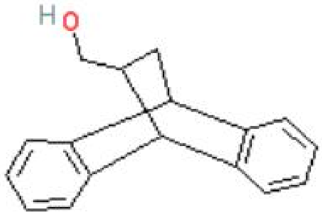	$C_{17}H_{16}O$	No	236.3	n/a	NCI	Yes	No	No	Yes (208,6)	?	A*	71	
1492-0330	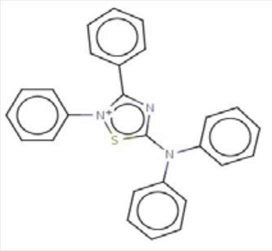	$C_{26}H_{20}N_3S$	No	406.5	5-(diphenylamino)-2,3-diphenyl-1,2$l^{5},4-thiadiazol-2-ylium	Chem Div	Yes	Yes (125)	No	Yes (143,3)	?	B/C*	72	

5107930	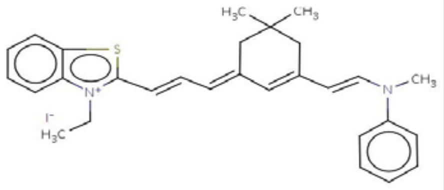	$C_{29}H_{33}IN_2S$	Yes	568.6	2-[(1E)-3-[(1E)-5,5-dimethyl-3-[(E)-2-[methyl(phenyl)amino]ethenyl]cyclohex-2-en-1-ylidene]prop-1-en-1-yl]-3-ethyl-1,3-benzothiazol-3-ium iodide	Chem Bridge	Yes	Yes (31,25)	Yes (125)	Yes (27,9)	?	B/C*	73	plate 8
4090-1979	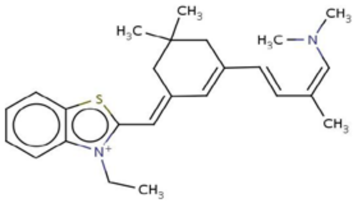	$C_{25}H_{33}N_2S$	Yes	393.6	n/a	Chem Div	Yes	Yes (62,5)	No	Yes (46,2)	?	B/C*	74	
1493-0289	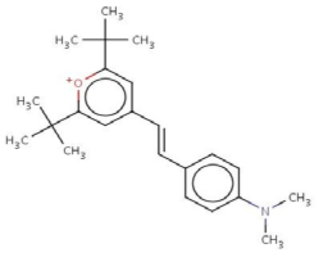	$C_{23}H_{32}NO$	Yes	338.5	2,6-di-tert-butyl-4-[(E)-2-[4-(dimethylamino)phenyl]ethenyl]-1$l^{4}-pyran-1-ylium	Chem Div	Yes	Yes (3,9)	Yes (125)	Yes (6,8)	?	B/C*	75	
BAS 00127538	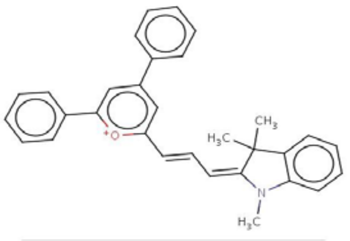	$C_{31}H_{28}NO$	Yes	430.6	2,4-diphenyl-6-[(1E)-3-[(2E)-1,3,3-trimethyl-2,3-dihydro-1H-indol-2-ylidene]prop-1-en-1-yl]-1$l^{4}-pyran-1-ylium	Asinex	Yes	Yes (1,95)	Yes (7,8)	Yes (2,7)	?	B/C*	76	
						**All concentrations are in µM**							total amount 75 + DMSO	

*Compound category	
A	**Not effective compound**<br>Not active against S.aureus<br>Not active or active against E.coli<br>Not toxic or toxic for tested cell lines
B	**Effective but not specific**<br>Highly active against S.aureus<br>Highly active against E.coli<br>Toxic for tested cell lines
C	**Effective and specific, antibiotic candidate**<br>Highly active against S.aureus<br>less active against E.coli<br>Not (strong) toxic for tested cell lines
*	Lipid II binding

$IC_{100\%}$ Inhibitory concentration on which 100% killing of bacteria occured, determinded by no bacterial regrowth after exposure to the compound
